# The HIV Empowering Adults’ Decisions to Share: UK/Uganda (HEADS-UP) Study—A Randomised Feasibility Trial of an HIV Disclosure Intervention for Young Adults with Perinatally Acquired HIV

**DOI:** 10.1007/s10461-024-04294-2

**Published:** 2024-03-15

**Authors:** Michael Evangeli, Georgina Gnan, Victor Musiime, Sarah Fidler, Janet Seeley, Graham Frize, Annette Uwizera, Matteo Lisi, Caroline Foster

**Affiliations:** 1 Department of Psychology, Royal Holloway University of London, Egham, Surrey, TW20 0EX UK; 2 Makerere University, Kampala, Uganda; 3 Joint Clinical Research Centre, Kampala, Uganda; 4 Department of Infectious Disease, Imperial College London, Imperial College NIHR BRC, London, UK; 5 London School of Hygiene and Tropical Medicine, London, UK; 6 Central and North West London NHS Foundation Trust, London, UK; 7 Imperial College Healthcare NHS Trust, London, UK

**Keywords:** HIV, Disclosure, Perinatal, Feasibility, Intervention

## Abstract

Young adults with perinatally acquired HIV (PAH) face numerous challenges, including antiretroviral therapy (ART) adherence, managing onward HIV transmission risks and maintaining wellbeing. Sharing one’s HIV status with others (onward HIV disclosure) may assist with these challenges but this is difficult. We developed and tested the feasibility of an intervention to help HIV status sharing decision-making for young adults with PAH. The study used a randomised parallel group feasibility design with 18–25-year-olds in Uganda and 18–29 year-olds in the UK. Participants were randomly assigned to intervention or standard of care (SOC) condition. The intervention consisted of four sessions (3 group, 1 individual) with follow-up support, delivered in person in Uganda and remotely in the UK. Assessments were carried out at: Pre-intervention /baseline; Post-intervention (intervention group only); Six-month follow-up. 142 participants were recruited (94 Uganda, 48 UK; 89 female, 53 male). At six-month follow-up, 92/94 (98%) participants were retained in Uganda, 25/48 (52%) in the UK. Multivariate analysis of combined data from both countries, showed a non-significant effect of intervention condition on HIV disclosure cognitions and affect (p = 0.08) and HIV disclosure intention (p = 0.09). There was a significant intervention effect on well-being (p = 0.005). This study addressed important gaps in understanding acceptable and feasible ways of delivering HIV status sharing support for young people living with PAH across two very different settings. The intervention was acceptable in both countries and feasible in Uganda. In the UK, retention may have been affected by its remote delivery.

Trial registration: ISRCTN Registry, ISRCTN31852047, Registered on 21 January 2019.

**Supplementary Information:**

The online version contains supplementary material available at 10.1007/s10461-024-04294-2.

## Introduction

Globally, an estimated five million young people (15–25 years) live with HIV, with a significant proportion having acquired HIV perinatally (Perinatally Acquired HIV: PAH) [1]. There are an estimated 170,000 15–24 year olds living with HIV in Uganda [2] (many of whom live PAH) with an HIV prevalence in this age range of 2.9% in females and 0.8% in males [3]. The UK has a small number of people living with PAH [4]. A total of 2,212 children were ever reported to the UK Collaborative HIV Paediatric Study by the end of March 2021 [5]. The majority of the UK cohort is of sub-Saharan African origin, with half born outside the UK [6].

Although young people living with PAH share some similar challenges to those who are behaviourally infected (e.g., adherence to lifelong antiretroviral therapy (ART), potentially compromised health, HIV stigma, risk of onward HIV transmission), other stressors relate specifically to perinatal acquisition. Longstanding HIV infection acquired prior to physiological and immunological development results in chronic clinical complications during childhood that can cause severe morbidity [7]. In addition to dealing with chronic illness and its associated stressors (e.g., hospitalisations, missed school and social opportunities, and pain), young people living with PAH have often experienced multiple caretaking transitions and loss due to parental and/or sibling illness or death. Young adults with PAH have often additionally experienced suboptimal ART regimens, increasing their likelihood of drug resistance [8].

Daily oral ART medication can transform survival for people living with HIV and prevent the risk of onward transmission to partners and infants. However, ART-mediated viral suppression requires daily adherence to medication and is challenging for some. Rates of viral suppression are variable globally in young people living with PAH, with the lowest rates among 13–24-year-olds when compared to younger children or older adults [8]. Only 54.7% of Ugandan 15–24 year olds are virally suppressed, the lowest of any age range [3]. For many young adults, regardless of their HIV status, negotiating their first sexual experiences can be complex, with the importance of condom use varying depending on cultural and religious norms [9, 10]. For young people with PAH, sexual onset will occur alongside knowledge of having a sexually transmittable, stigmatised medical condition and the potential for onward transmission in those not on suppressive ART.

Sharing an HIV-positive status with others (onward HIV disclosure) has the potential to facilitate positive outcomes in the above areas. It is proven that successful viral suppression (< 200 copies HIV RNA/ml) with ART prevents HIV transmission to sexual partners (Undetectable = Untransmittable; U = U) [11, 12]. In situations where viral suppression has not been achieved, however, sharing an HIV-positive status with partners may reduce onward HIV transmission, by fostering communication about safer sex strategies. HIV status sharing may encourage a partner to undergo testing, use prevention strategies such as condoms, pre-exposure prophylaxis (PrEP) and post-exposure prophylaxis (PEP), and engage in care/treatment if needed [13].

HIV status sharing has also been shown to have a number of *personal* benefits [14]. Sharing may help to buffer HIV-related stress and improve wellbeing [15, 16]. Being open about one’s HIV status may facilitate obtaining social support from significant others, which in turn may assist constructive coping, enhance self-esteem and other health-promoting behaviours [16]. Sharing one’s HIV status may also enhance ART adherence, due to greater adherence support from partners, friends or family, and a reduced need to hide medication use in situations where sharing has not occurred [17, 18]. This is important even for young adults who have an undetectable viral load, as they will only remain undetectable if they continue to adhere to ART. Fear of sharing HIV status is commonly cited as a barrier to ART adherence in young people living with PAH [19]. There is also evidence of lower levels of HIV status sharing being associated with poorer engagement with HIV care [20–22]. There is no direct evidence of a relationship between HIV disclosure and unprotected sex in those with PAH. There is, however, evidence in other people living with HIV [23, 24]. Lower levels of status sharing, however, has been associated with more partners in a PAH sample [25].

Globally, there are variable rates of self-reported sharing of an HIV-positive status with a partner, ranging from 39 to 97% [26]. These rates appear to be particularly low in young people with PAH. Mugo and colleagues [27] reported that 66% of their sample of youth living with HIV either married or sexually active in Kenya had not shared with their partners. Only 40% of young adults living with PAH in the US reported sharing with all or most of their partners in one study, and 45% reported sharing with no partners when having unprotected sex [13]. There are also low rates of HIV sharing to other (non-partner) members of one’s social network. In a study in Uganda and Kenya only one in five participants aged 13–17 years reported having shared their HIV status with their peers and almost half had told nobody (except health-care providers) about their HIV status [16].

Sharing an HIV status carries a unique challenge for young people with PAH, with concerns about revealing their mother’s and potentially other family members’ HIV status [28]. Negative parental sharing attitudes, including directives to not share, may be internalised [28] with an atmosphere of secrecy and limits to open communication about HIV affecting the young person at home, in their community and in the clinic [29, 30]. The subjective difficulty of sharing one’s HIV status in young people with PAH, particularly in relationships, has been frequently reported, with a fear of rejection, a lack of confidence about sharing (low HIV disclosure self-efficacy), and fear of secondary disclosure from the recipient to others, cited as barriers [28, 31–34]. There remain important risks of sharing ones’ HIV status, including the threat of rejection, humiliation, stigma and violence [13, 35].

There are fewer reports in the literature relating to facilitators of HIV status sharing. Viljoen et al. [36] reported that trust and intimacy can motivate sharing. HIV status sharing in young people with PAH has been associated with higher levels of HIV disclosure self-efficacy, being older, paternal orphanhood, contributing to family income, regular visits to the HIV clinic, and greater social support through peers [16]. Status sharing has also been associated with females, earlier age of naming (also known as paediatric HIV disclosure, the process of informing children or adolescents of their HIV diagnosis), increased STD knowledge, HIV disclosure intentions, parent–child communication [13] and with lower levels of HIV stigma and depression [27].

Given the potential benefits of sharing ones’ HIV status, there have been efforts to develop multi-session HIV disclosure interventions in other populations of people living with HIV. Serovich and colleagues developed an HIV disclosure intervention for MSM sharing their HIV status with different recipients, for example family members and partners [37–40]. Across studies, the intervention has not been found to increase HIV disclosure compared to participants in attention control conditions [38–40]. The Amagugu intervention aims to assist in maternal HIV disclosure to a child not living with HIV. The intervention is a home-based, lay-counsellor led intervention and has been shown to be effective in increasing mothers’ confidence in their ability to disclose, and increasing disclosure rates compared to baseline rates [41]. The Teach Raising and Communicating with Kids (TRACK) intervention aims to increase mothers living with HIV (MLH) sharing their status with their children. The authors [42] found that participants who took part in the intervention were four times more likely to disclose their status to their children than those in the wait-list control condition.

Aside from stand-alone multicomponent interventions, other approaches to enhancing HIV status sharing have included training community health workers [43], presenting video material [26], support groups, involving peer workers and different methods of partner notification [44]. There are no HIV status sharing interventions specifically designed for young people with PAH, or young people with HIV more generally [45]. However, facilitating onward disclosure has been a small component of multi-component interventions for young people living with HIV [46–49].

In standard care there is evidence of counselling for adolescents living with HIV stressing the benefits of HV sharing and not acknowledging fears and risks [50]. Consistent with the gaps in the evidence base outlined above, there is a lack of HIV status sharing guidance to support young people with PAH or professionals working with this population [45]. The World Health Organisation (WHO) has called for work in this area, specifying the need for interventions to help adolescent disclosure decision-making, support caregivers and train providers [51]. There is evidence that young people with HIV [52–54] and health care workers [55] would like more HIV status sharing support.

We aimed to develop and test the feasibility of a behavioural intervention to empower young adults with perinatally acquired HIV in the UK and Uganda to make decisions about HIV status sharing. The appropriateness of the intervention in high income/low prevalence (e.g., UK) and low income/high prevalence (e.g., Uganda) contexts needs to be assessed, given global evidence of low rates of HIV status sharing. The decision to focus on young adults rather than a younger population was due to higher rates of sexual activity in the former, with potentially more active consideration of sharing than in younger populations, as well as higher mortality and morbidity in this age group [56]. In addition, in this age range, decisions about sharing may be less constrained by one’s family than during earlier adolescence. The novel focus was on sharing with *any* recipient depending on participant preference. Enhancing sharing with one category of recipient could facilitate sharing with other categories. For example, increased HIV status sharing and communication with friends and family has been shown to be associated with sharing with a partner [13, 33].

We hypothesised that (1) the intervention would be feasible, in relation to recruitment, retention and acceptability (primary study outcome) and (2) participants in the intervention group will have more pro HIV sharing motivation and intention, and higher levels of well-being at follow-up than participants in a standard of care (SOC) group (secondary study outcome).

## Methods

### Design

**Fig. 1:**
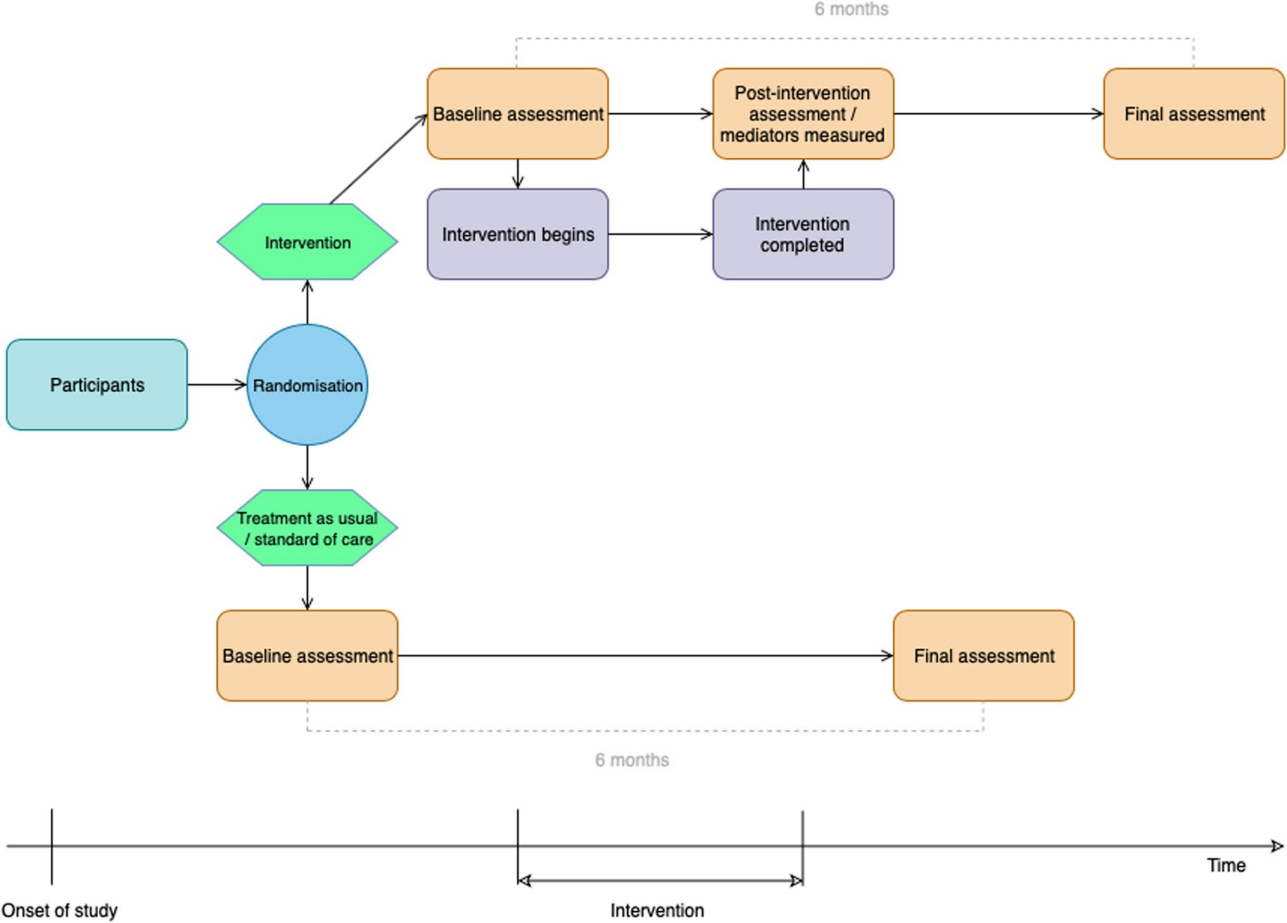
Study design

The study used a parallel randomised feasibility design. Participants were randomised to either intervention or SOC condition. Assessments were carried out at pre-intervention/baseline (for both conditions), post-intervention (at the end of the final session, only for the intervention condition), and six-month follow-up (six months from baseline, both conditions). See Fig. 1 for a diagram of the study design.

### Setting

The study took place in two countries:

1. UK. Participants were recruited from six inner city NHS clinics providing services for young people living with PAH across three cities, as well as from one UK-based national HIV charity.
2. Uganda. Participants were recruited from a not-for-profit organisation in one city, providing HIV care to young people living with PAH ≥ 18 years.

### Inclusion and Exclusion Criteria

#### Inclusion Criteria

Participants were included if they were aged 18 to 25 years inclusive in Uganda and 18 to 29 years in UK, living with PAH, receiving HIV care at study sites/recruitment sources, and aware of HIV status. The age range was larger in the UK to maximise recruitment.

#### Exclusion Criteria

Participants were excluded if they had current serious mental health problems, moderate to severe learning disability/executive functioning difficulties, current serious physical health problems with life expectancy < 12 months, unable to understand or communicate in English (UK) or either English or Luganda (Uganda), currently participating in other psychosocial intervention/support research (UK), had participated in the intervention development phase of the study [57], or had another individual in the household who had participated in this phase of the study.

### Sample

142 participants were recruited (94 Uganda, 48 UK), a sample size consistent with existing guidance for pilot studies [58]. See Tables 1 and 2 for demographic and clinical characteristics. Enrolment took place between 9th December 2020 and 15th March 2022.

**Table 1:** Sample demographic characteristics (n = 141 unless stated)

	Frequency (%)
Gender	
Male	53 (37.6)
Female	88 (62.4)
Age (in years)	
Mean (SD)	22.9 (2.6)
UK ethnic group (n = 34)	
Black	28 (82.4)
Asian	2 (5.9)
Mixed	1 (2.9)
Other	3 (8.8)
Uganda tribe (n = 93)	
Baganda	63 (68)
Banyankore	6 (6.5)
Basoga	4 (4.3)
Bakiga	4 (4.3)
Bagisu	3 (3.2)
Iteso	3 (3.2)
Other	10 (10.8)
Relationship status (n = 127)	
Single	77 (60.6)
Relationship, living together	13 (10.2)
Relationship, not living together	37 (29.1)
Living situation (n = 127)	
Alone	22 (17.3)
With family	87 (68.5)
With friends	4 (3.1)
With partner	12 (9.4)
With family and others	2 (1.6)
Biological parents (n = 125)	
Both alive	44 (35.2)
Father not alive	24 (19.2)
Mother not alive	33 (26.4)
Neither parent alive	21 (16.8)
Don’t know	3 (2.4)

**Table 2:** HIV and clinical characteristics at baseline

	Frequency (%)
Age of paediatric HIV disclosure/naming (n = 122)	
< 10 years	31 (25.4)
10–12 years	54 (44.3)
> 12 years	37 (30.3)
Lifetime HIV disclosure (number disclosed to)(n = 123)	
0	17 (13.8)
1	11 (8.9)
2	15 (12.2)
3	18 (14.6)
4–9	32 (26.0)
10+	30 (24.4)
HIV disclosure in last 6 months (number disclosed to—direct disclosure or indirect with permission) (n = 127)	
0	113 (89.0)
1	6 (4.7)
2+	8 (6.3)
Proportion of social network aware of HIV status (n = 123)	
Median % (IQR)	75 (50–100)
Sexual behaviour	
Sexual activity in last 6 months (n = 111)	65 (58.6)
Sexual activity in last 4 weeks (n = 111)	45 (40.9)
Last partner’s HIV status (n = 55)	
Positive	8 (14.5)
Negative	36 (65.5)
Not known	11 (20)
Last partner condom use (n = 91)	
Yes	49 (53.8)
No	42 (46.2)
On ART (n = 127)	
Yes	124 (97.6)
No	3 (2.4)
Viral load (copies/mL) (n = 125)	
< 200	116 (92.8)
> 200	9 (7.2)

### Intervention

The intervention consisted of four-sessions: three group sessions, consisting of a maximum of eight participants, and one individual session. The groups sessions were led by one professional (psychosocial counsellor, clinical nurse specialist, and social worker) and one peer worker. In Uganda the sessions were conducted by bilingual therapists (Luganda and English). Each therapist carried out individual sessions. Therapist training (covering counselling skills, running groups and the intervention) was carried out over eight half days by the first author remotely. The groups were mixed gender. Follow-up support was offered by the peer worker in the six months from baseline to the follow-up data point for both participants and their social network.

The intervention aimed to develop motivation and skills for HIV status sharing, decrease anxiety about HIV sharing and decrease decisional conflict about HIV sharing decision-making. It used a Motivational Interviewing (MI) structure [59]. That is, the first session focused on engaging with participants element before focusing on HIV sharing and evoking motivations to share or not to share in subsequent sessions. The intervention was carried out remotely in the UK due to the COVID-19 pandemic, and in person in Uganda.

**Fig. 2:**
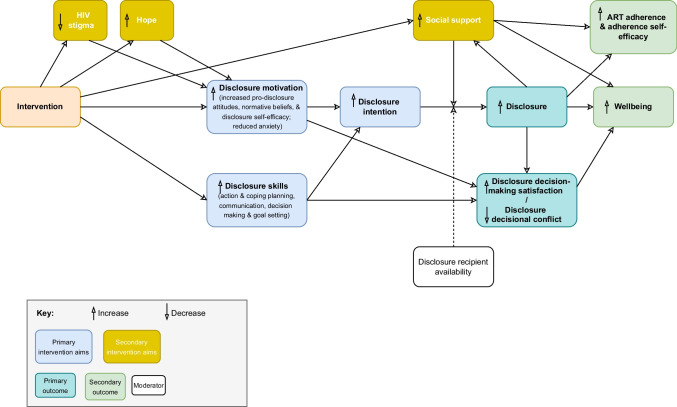
Intervention conceptual model

The intervention was based on a conceptual model (see Fig. 2), which in turn was based on models of HIV disclosure decision-making and HIV disclosure anxiety [60–63] and existing evidence of HIV status sharing correlates, barriers and facilitators [13, 16, 64]. In particular, disclosure attitudes, normative beliefs, self-efficacy, planning and support were hypothesised to be important proximal determinants of HIV status sharing. These factors are suggested to be influenced by more distal factors: personal values and HIV stigma.

The components of specific sessions were as follows:

•Session 1—*Engaging*: ice breaker; intervention aims; ground rules; living with HIV; what do I need to know about HIV to be ready to share?; what is important to me (values clarification exercise)?

•Session 2—*Focusing and Evoking*: HIV sharing quiz; what reasons are there for not sharing or sharing an HIV status; anxiety about sharing; video—experiences of sharing; personal guidelines about sharing.

•Session 3—*Developing HIV Status Sharing Skills*: Dramatized video of HIV sharing; If you’ve decided to share to a particular person—where, when and how to share, and what to say; practising sharing; after sharing.

•Session 4—*Goal Setting and Planning*: Developing a personal sharing plan: assessing goals including reasons for goal and relationship between goal and personal values; developing an action and coping plan. Participants were encouraged to keep a copy of their plan to refer to in the future.

Exercises used role play, modelling, and cognitive restructuring techniques, with both video material and group discussions integrated with interactive exercises. Some components (e.g., values clarification and identifying values-consistent sharing goals) were novel. Each participant was given a workbook to accompany the intervention. The key characteristics of the intervention were adapted for each study context based on earlier formative work, although the core elements of the intervention were the same in each setting. The sessions were one week apart to allow reflection on HIV sharing to take place between sessions. Participants were allowed to move on to the next session if they had missed a previous one.

### Intervention Fidelity

All intervention sessions were taped. Ten sessions in each country were reviewed by the one of the research team (after acceptable inter-rater reliability between the research team had been established by jointly rating sessions) for fidelity to the intervention. An intervention checklist and a generic therapy competence tool were used for this task. Feedback was offered to the therapists based on this review, as well as problem-solving supervision. The criteria for acceptable performance were to have been rated as having ‘done well’ on at least three of the following general therapy competencies: Non-verbal communication & active listening; Verbal communication skills; Rapport building & self-disclosure; Exploration, interpretation & normalisation of feelings; Demonstration of empathy, warmth & genuineness, and to have `done’ or `done to some extent’ at least 80% of session activities.

Additional strategies to enhance fidelity included therapist training and the use of an intervention manual (see Supplemental Material 1). In addition, therapists monitored each session that they had facilitated using an intervention session checklist, which was reviewed in supervision. Treatment receipt [65] was assessed in session evaluation and acceptability questionnaires given to participants with questions such as, *“How easy was the session to follow?”* and “*How much effort did it take to participate in the intervention?”* with responses recorded on seven-point Likert scales. Treatment enactment [65] was measured in the acceptability questionnaire by asking, *“How effective was the intervention in helping you to make decisions about sharing your status?”* with responses on a seven-point Likert scale.

### Standard of Care Condition

The standard of care condition was routine care. The UK NHS sites, and the Uganda site had dedicated clinics for young people growing up with HIV who have previously been in paediatric care, with peer support and professional psychosocial support available in the Uganda site and in the majority of the UK NHS sites. In these clinics, young people could discuss HIV sharing with their multidisciplinary team, attend with their partner, and have access to a range of other services (e.g., HIV testing, PrEP, family planning, ART adherence support and condom provision). Standard of care in both countries, however, was for there to be no routine or structured psychosocial intervention to facilitate HIV sharing or sharing decision-making. To assess standard of care, participants were asked, “*Have you spoken to anyone about sharing your status in the last 6 months?*” at the follow-up stage. If yes, they were asked if this was part of their usual care.

### Ethics

Ethical approval was granted by a UK NHS research ethics committee and the UK Health Research Authority, and by Royal Holloway University of London Research Ethics Committee. Local approval in the UK was granted by NHS research and development departments. In Uganda, ethical approval was granted by the Joint Clinical Research Centre Ethics Committee and Uganda National Council of Science and Technology. Informed written consent was obtained for all participants.

### Measures

#### Primary Study Outcome

The primary outcome of the study was its feasibility. This was assessed by in relation to recruitment, retention and acceptability. Post intervention acceptability was assessed for participants in the intervention condition by rating 12 items using seven-point Likert scales. An example question was, *“How do you feel about the intervention?”* A total score was calculated with a range of possible scores of 12 to 84 (α = 0.73). Participants were also asked to rate each session through eight questions (e.g., *“How much did you enjoy this session?*”) using seven-point Likert scales (α = 0.75). A total score was calculated with higher scores indicating greater levels of acceptability (range of possible scores: 8–56). They were also asked an open question for any comments.

The perceived efficacy of the intervention was assessed for those allocated to the intervention condition in the following way:

1. Participants were asked, *“Have you spoken to anyone about sharing your status in the last 6 months?”* at the follow-up stage. If yes, they were asked if this was due to taking part in the project.
2. Participants were asked the following questions at the post-intervention point (answered on 7-point Likert scales):
  - *How effective was the intervention in helping you to make decisions about sharing your status?*
  - *How likely is the intervention to be helpful to others in making decisions about sharing their status?*
  - *How much do you intend to use things that you learned in the intervention in the future?*

Social harms potentially associated with the intervention were assessed in two main ways:

1. An item on the follow-up demographic questionnaire, *“Did anything negative happen as a result of taking part in the project (for example, problems with friends, family, partner; feeling lower)? If yes, please state.”*
2. An item on the follow-up disclosure behaviour questionnaire for each disclosure recipient, *“How much do you agree with the following statement: Overall they responded positively when they found out that I was HIV positive.”* Responses were on a five-point Likert scale from *strongly disagree* to *strongly agree*. This question was asked if the participant had shared their status in the last six months.

#### Secondary Study Outcome Measures

Secondary study outcomes related to whether the aims of the intervention (e.g., increasing HIV disclosure motivation, intention and behaviour) were met. Scales in English were translated into Luganda during the intervention development phase and adapted as necessary [66]. Participants in Luganda were given dual language versions of the measures.

##### HIV Disclosure Behaviour

HIV disclosure behaviour was assessed through recording (a) the frequency of new disclosure events (first hand or second hand with consent) in the last six months to partners, friends and family, and (b) the proportion of participants’ social network who knew the HIV status of the participant (number of individuals in the social network aware of HIV status divided by the total number in the social network × 100).

Participants were asked to list who was in their social network and then, for each person, were asked a series of questions relating to HIV sharing (e.g., “*Do they know that you are HIV positive? How long have they known? Did you tell them yourself?”*).

##### Wellbeing

The 6-item psychological domain from the *World Health Organisation Quality of Life brief questionnaire* (WHOQOL BREF) [67] was used. This measure has been translated into Luganda with good evidence of reliability and validity [68]. It has also been extensively used with people living with HIV, including young adults with HIV in South Africa [69]. It includes questions on bodily image and appearance, negative feelings, self-esteem, spirituality/religion/personal beliefs, thinking/concentration, and positive feeling, (e.g., “*How much do you enjoy life?”*) which are answered on 5-point scales (e.g., from 1-*not at all* to 5-*completely*) relating to the last four weeks (in this study, α = 0.82 at BL; α = 0.78 at f-up).

##### Social Support

The 6-item *Social Support Questionnaire Short form*—SSQ6 [70] was used. For each of the 6 items (e.g., *whom can you really count on to help you feel more relaxed when you are under pressure or tense?),* respondents indicate the number of people available to provide support and then rate the overall level of satisfaction with the support given in each of the areas from 6-*very satisfied* to 1-*very dissatisfied*. Internal consistency in this study was as follows: α = 0.93 at BL; α = 0.90 at f-up. A longer form of this measure has been used with young people living with HIV in Uganda [71].

##### Hope

The 6-item State Hope Scale was used [72] as a self-report measure of ongoing goal-directed thinking. It includes items such as “*There are lots of ways around any problem that I am facing now*” and is scored on an 8-point Likert scale from 1 (*definitely true*) to 8 (definitely false). This measure has been used with young adults in South Africa [73]. In the current study, α = 0.79 at BL; α = 0.79 at f-up.

##### Decisional Conflict

The 4-item Decisional Conflict Scale was used in relation to decisions to share one’s HIV status with others [74]. The four items are: feeling uncertain (Sure of myself), feeling informed (Understand information), feeling clear about values (Risk–benefit ratio), and feeling supported in decision making (Encouragement). A response of yes scores 1 and a response of no scores 0. Higher total score indicated greater decisional conflict. In this study alpha for the total score was 0.60 at BL and 0.62 at f-up.

##### HIV Disclosure Cognitions and Affect

*The Adolescent HIV Disclosure Cognition and Affect Scale (AHDCAS)* [75] was used. This 18-item scale measures negative disclosure attitudes and feelings, positive disclosure attitudes and feelings and disclosure self-efficacy, and has demonstrated good reliability (α = 0.79) and validity in a sample of UK adolescents living with PAH. An example item is “*I am confident that I can deal with how others respond if I share my HIV status with them*”. Responses are made on a five-point Likert scale from (1) *strongly disagree* to (5) *strongly agree*. The minimum score is 18 and the maximum is 90, with higher scores indicating more positive sharing attitudes, feelings about sharing, and sharing self-efficacy. Internal consistency in this study was: α = 0.81 at BL; α = 0.81 at f-up. An additional HIV disclosure intention item, “*I intend to tell someone new about my HIV status in the next 6 months*”, using the same response options, forms the complete measure. Higher scores indicate greater intention to disclose.

##### HIV Disclosure Planning Specificity

HIV disclosure planning specificity was assessed by asking the following, relating to action and coping planning: *“If you wanted to share your HIV status with someone who did not know your status: (a) How would you do this? (b) How would you respond to what they might say?”* Responses were rated on a 0 to 2 scale, with 0 indicating low specificity and 2 indicating high specificity. These questions and the same method of coding responses have been used with adolescents with PAH in the UK [76].

##### HIV Stigma

The three-item negative self-image subscale from the short form of the HIV stigma scale was used [77]. An example item is “*I feel I’m not as good a person as others because I have HIV*”, and responses are on a 4-point Likert scale, ranging from *strongly disagree* (1) to *strongly agree* (4) (α = 0.74 at BL; α = 0.77 at f-up). The HIV stigma scale has been used extensively, including in young people living with HIV in South Africa [78].

#### Measurement of Background Variables

##### Previous HIV Disclosure

Participants were asked about lifetime HIV sharing frequency with the following question – *“How many people have you told about your HIV status?”.* The following response options were offered: 0, 1, 2, 3, 4–9. 10+.

##### Demographic and Clinical Variables

Demographic and background variables (current age, ethnic group/tribe, gender, age of paediatric disclosure/naming, parental loss, relationship status, living situation, sexual behaviour, education/occupation) and clinical variables (ART, viral load) were assessed. ART and viral load information was obtained from clinic records.

### Procedure

Clinicians or research coordinator approached potentially eligible individuals with study details and took informed consent. After enrolment, stratified block random allocation to condition by country was used, with equal numbers allocated to each condition. Allocation was concealed from the study coordinator in each country. Assessments were conducted remotely via Qualtrics survey software in the UK and mostly in person in Uganda. In Uganda, the measures were administered in a face-to-face group format. Participants read each question and recorded their responses individually. Travel expenses were paid to participants in both the intervention and the SOC conditions. Participants were also reimbursed for completing study measures (UK—£20 at baseline and follow-up, £10 at post-intervention: Uganda—50,000 Shilling at baseline and follow-up, 25,000 Shilling at post-intervention).

Methods used to maximise attendance included communicating about intervention sessions in advance, timetabling sessions at regular times, giving participants the opportunity to meet with a study therapist before the intervention started, offering prompts (Whatsapp and/or phone) and following up when participants did not attend.

### Analysis Plan

After descriptive analysis, we conducted multivariate analyses to estimate intervention effects at the six-month data point on three dependent variables: AHDCAS, HIV Disclosure Intention, and WHOQOL-BREF. In all cases we used multilevel (mixed-effects) models to account for the longitudinal design of the study. In the case of HIV Disclosure Intention, the dependent variable consisted of only a single item with five ordered response options. Therefore, we used a multilevel ordinal logistic regression model. The remaining dependent variables were analysed with linear multilevel models, using Satterthwaite approximation to find effective degrees of freedom and compute p-values. In the case of linear models we examined the distribution of residuals to confirm that it did not display obvious deviation from normality. All models, linear or ordinal, had the same random-effects structures, consisting of random, subject-specific intercepts, that accounted for the heterogeneity across individuals in each dependent variable. In all models the main fixed-effects predictors were the intervention condition (intervention vs SOC) and the timepoint (baseline vs follow-up). Additionally, the models included several covariates. For the analyses of the AHDCAS and HIV Disclosure Intention, we included the following covariates: gender, age, country (UK vs Uganda), and lifetime HIV disclosure at baseline (i.e., the total number of people disclosed to). For HIV disclosure intention, we included age of naming. For categorical covariates (gender and country) we used sum contrasts, also known as deviation coding. Continuous covariates (age and lifetime HIV disclosure) were standardized (i.e., centred and scaled by their standard deviation). For analyses with WHOQOL-BREF as the dependent variable we included only gender, age and country as covariates.

The AHDCAS had a large fraction of missing values (31% at baseline and 30% at follow-up) due to participants failing to complete all the scale items. To account for these missing data within our dataset, we conducted an additional imputation analysis using multiple imputation. The results of the models fit on the imputed datasets were consistent with the complete case analyses and revealed the same set of significant predictors (see Supplemental Material 2 for a more detailed report of the imputation analysis).

## Results

### Primary Study Outcomes

#### Recruitment and Retention

**Fig. 3:**
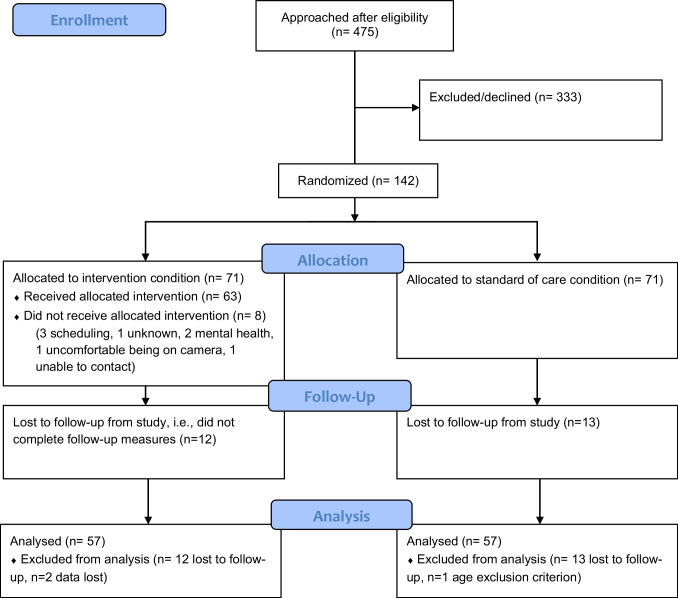
CONSORT diagram

The initial recruitment target of 94 participants in each country was reduced in the UK due to recruitment difficulties. 142 participants were recruited (94 Uganda, 48 UK; 89 female, 53 male). At six-month follow-up, 92/94 (98%) participants were retained (i.e., completed measures) in Uganda, 25/48 (52%) in the UK. 59/71 (83%) participants were retained from the intervention condition, 58/71 (82%) participants were retained from the SOC condition (See Fig. 3).

Sixty-three participants out of 71 (89%) allocated to the intervention condition attended at least one session. 51/71 (72%) attended all four sessions (and 57/71 (80%) attended either 3 or 4 sessions). The median session length was 112 min (group) and 28 min (individual).

### Intervention Acceptability, Use and Perceived Impact

The total score on the intervention acceptability measures (maximum score 84) showed high levels of acceptability in both countries; overall mean 73.18 (sd 6.04); Uganda 73.02 (sd 5.77); UK 73.90 (sd 7.49). The total score on the session evaluation questionnaire (maximum score 56) was also high (i.e., very positive) in both countries: overall median 53 (IQR 49–55); Uganda 52 (IQR 49–55); UK 55 (IQR 52–56).

The sharing recipient identified for the session four goal was partner 29 (50%), friend 14 (24%), family 14 (24%), everyone 1 (2%). Follow-up support was rarely used, with only four participants in Uganda and one participant in the UK contacting the peer worker after the intervention sessions. Thirty eight out of 56 (68%) intervention participants said that they had spoken to someone about HIV sharing in the last six months because of the project. Participants rated the intervention as effective in helping with decisions about sharing (mean 6.29, sd 0.85); likely to be helpful to others (mean 6.23, sd 0.81); and stated that they intended to use the intervention in the future (mean 6.29, sd 0.78).

Nine out of 58 (16%) participants stated that something negative had happened due to taking part in the project. Of these nine, only one indicated a negative impact on their mood. One said that making it to the session was hard. Two said that they were not believed after sharing their status. One said that they were rejected after sharing. Four did not specify the reason for something negative. Eight Uganda participants (5 intervention, 3 SOC) were referred on to other professionals (6 to counsellors, 2 to doctors) over the course of the study. Those referred to doctors has questions regarding their treatment. In relation to assessing HIV disclosure discussions in usual care, 15/54 (28%) in the SOC condition had spoken to a health professional (doctor, nurse, or counsellor) about HIV sharing in the previous 6 months.

### Secondary Study Outcomes

#### Descriptive Analysis

Descriptive scores on measures by condition at different time points are outlined in Table 3.

**Table 3:** Descriptive statistics for measures

Measure	Total or subscale name (possible range of scores)	Baseline Mean (sd)		Post-intervention Mean (sd)	Follow-up Mean (sd)	
		Intervention	SOC	Intervention	Intervention	SOC
The Adolescent HIV Disclosure Cognition and Affect Scale	Total Scale (18–90)	56.16 (11.22)	57.96 (10.81)	63.49 (9.58)	61.00 (11.44)	59.90 (11.11)
HIV Disclosure Intention	Intention score (1–5)	2.76 (1.52)	2.88 (1.42)	3.64 (1.18)	3.22 (1.44)	2.69 (1.44)
HIV Disclosure in last 6 months (direct or indirect disclosure with permission)	No disclosure	58/66 (87.9%)	55/61 (90.2%)		42/45 (93.3%)	34/36 (94.4%)
WHOQOL—BREF	Total score (6–30)	22.33 (4.37)	22.74 (5.60)	24.40 (2.99)	22.98 (4.24)	21.65 (4.62)
HIV Stigma Scale	*Negative self-image subscale* (3–12)	6.16 (2.31)	6.16 (2.55)	5.35 (2.20)	6.07 (2.48)	5.80 (2.25)
Social Support Questionnaire Short Form—SSQ6	*Social support satisfaction subscale* (6–36)	29.81 (7.16)	30.12 (8.40)	31.36 (5.75)	31.94 (5.12)	30.68 (7.95)
State Hope Scale	Total hope score (6–48)	33.70 (8.14)	34.28 (9.20)	37.46 (7.16)	35.52 (7.70)	35.78 (8.60)
HIV disclosure planning specificity	Total score (0–2)	0—52.5%, 1 or 2 – 47.6%	0 – 51.0%, 1 or 2 – 49%	0 – 33.3% 1 or 2 – 66.6%	0 – 32.7% 1 or 2 – 67.2%	0 – 55.0% 1 or 2 – 44.0%
CASE adherence	Total score (3–16)	11.48 (3.07)	11.81 (3.27)		12.00 (3.22)	11.65 (3.77)
Decisional Conflict Scale	Total score (0–4)	0.85 (1.06)	0.92 (1.17)	0.37 (0.60)	0.45 (0.80)	0.48 (0.95)

Several measures showed marked improvements in the intervention group at the end of the intervention, with scores falling back to levels between baseline and post intervention at six-month follow-up (AHDCAS, HIV Disclosure Intention, WHOQOL BREF, HIV Stigma Scale, and the State Hope Scale). The specificity of HIV disclosure plans improved in the intervention condition, and this was maintained at follow up. There was no evidence of greater HIV disclosure planning specificity in the SOC condition.

There was no evidence of a greater frequency of HIV disclosure in those allocated to the intervention condition, although the validity of our disclosure behaviour measure was unclear, and data should be considered with caution. The UK data at baseline and follow-up suggested very small social networks not consistent with the Uganda sample or with a previous UK study where the measure was administered by a researcher [79]. It may be that participants did not continue with the measure given its self-directed and online administration. It also became clear at the data analysis stage that Uganda participants in both conditions at follow-up were not asked about whether they had shared their status with anyone to whom they had not shared their status with at baseline. This error in administration further undermines the credibility of the measure of HIV disclosure behaviour.

Twenty-nine HIV disclosure events in the last six months were reported in the intervention arm at follow-up. In answer to the question, “*How much do you agree with the following statement: Overall they responded positively when they found out that I was HIV positive?*”, 22/29 (76%) responded agree or strongly agree with only 4/29 (14%) episodes (14%) answered strongly disagree or disagree.

#### Inferential Analysis

##### Adolescent HIV Disclosure Cognitions and Affect Scale

The results of this analysis are shown in Table 4. Overall, the only statistically significant predictor of AHDCAS was lifetime HIV disclosure at baseline. Each additional standard deviation in lifetime HIV disclosure at baseline (approximately 1.73 additional people to whom HIV status was disclosed) was associated on average with an increase of 4.59 in the AHDCAS score at both time points. There was a trend for the intervention to be associated with an increase in the ACHDAS score at follow-up relative to the SOC group, β = 4.22, 95% CI [-0.54, 8.97], p = 0.08. A similar pattern was observed when the same analysis was conducted after multiple imputation (see Supplemental Material 2).

**Table 4:** Results of the linear multilevel model used to analyse Adolescent HIV Disclosure Cognitions and Affect Scale

*Predictors*	**AHDCAS**				
	*β*	*95% CI*	*t*	*p*	*df*
(Intercept)	57.51	[54.14, 60.88]	33.72	** < 0.001**	141.95
Age	1.13	[− 0.92, 3.19]	1.09	0.278	98.79
Country	2.48	[− 0.03, 4.99]	1.95	0.053	98.83
Gender	− 0.08	[− 1.99, 1.83]	− 0.08	0.937	102.05
Lifetime Disclosure	4.59	[2.75, 6.44]	4.91	** < 0.001**	98.15
Time (follow up)	1.73	[− 1.48 to 4.95]	1.07	0.288	81.56
Condition (Intervention)	− 4.24	[− 8.58, 0.11]	− 1.93	0.056	155.81
Time (follow up) × Condition (Intervention)	4.22	[− 0.54, 8.97]	1.75	0.082	85.69
Random effects					
* σ2*	55.90				
* Τ*	50.87				
ICC	0.48				
N	107				
Observations	175				
Marginal R^2^/conditional R^2^	0.226/0.595				

Bold values indicate *p* ≤ 0.05

σ^2^ is the residual variance and τ the variance of the random intercepts. ICC is the intra-class correlation coefficient

#### HIV Disclosure Intention

The results of this analysis are shown in Table 5. We found a significant effect of lifetime HIV disclosure at baseline, indicating that participants who had disclosed to a larger number of people at baseline were on average also reporting higher HIV disclosure intentions at both time points. Additionally, we found significant effects of country, with Uganda participants reporting higher levels of HIV disclosure intentions than UK participants, and age of naming, with older age of naming associated with higher levels of HIV disclosure intention. There was a trend for the intervention to be associated with an increase in HIV disclosure intentions at follow-up relative to the SOC group, β = 0.95, 95% CI [-0.13, 2.04], p = 0.09.

**Table 5:** Results of the ordinal multilevel model used to analyse Disclosure intention

*Predictors*	Disclosure intention			
	*β*	*95% CI*	*z*	*p*
Age	0.21	− 0.19 to 0.61	1.05	0.294
Country	0.79	0.28 to 1.31	3.03	**0.002**
Gender	0.20	− 0.17 to 0.56	1.06	0.288
Lifetime Disclosure	0.46	0.11 to 0.82	2.57	**0.010**
Age of naming Time (follow up) Condition (Intervention) Time (follow up) × Condition (Intervention)	0.51 − 0.35 − 0.31 0.95	0.15 to 0.88 − 1.07 to 0.37 − 1.16 to 0.54 − 0.13 to 2.04	2.73 − 0.96 − 0.71 1.72	**0.006** 0.337 0.479 0.085
Random effects				
* σ2*	3.29			
* τ*	1.14			
ICC	0.26			
N	106			
Observations	199			

Bold values indicate *p* ≤ 0.05

#### WHOQOL-BREF

The analysis of the WHOQOL-BREF (Table 6) revealed several significant effects.

**Table 6:** Results of the linear multilevel model used to analyse WHOQOL-BREF

*Predictors*	WHOQOL-BREF				
	*β*	*CI*	*t*	*p*	*df*
(Intercept)	21.88	[20.54, 23.23]	32.14	** < 0.001**	149.93
Age	0.20	[− 0.69, 1.08]	0.44	0.663	113.97
Country	1.74	[0.65, 2.83]	3.16	**0.002**	117.23
Gender	− 0.06	[− 0.86, 0.75]	− 0.14	0.888	113.94
Time (follow-up)	− 1.06	[− 2.12, − 0.00]	− 1.99	**0.050**	100.84
Condition (Intervention)	− 0.45	[− 2.14, 1.23]	− 0.53	0.597	156.59
Time (follow-up) × Condition (Intervention)	2.22	[0.69, 3.76]	2.87	**0.005**	101.71
Random effects					
* σ*^2^	7.52				
* Τ*	13.30				
ICC	0.64				
N	118				
Observations	215				
Marginal R^2^/conditional R^2^	0.096 / 0.673				

Bold values indicate *p* ≤ 0.05

**Fig. 4:**
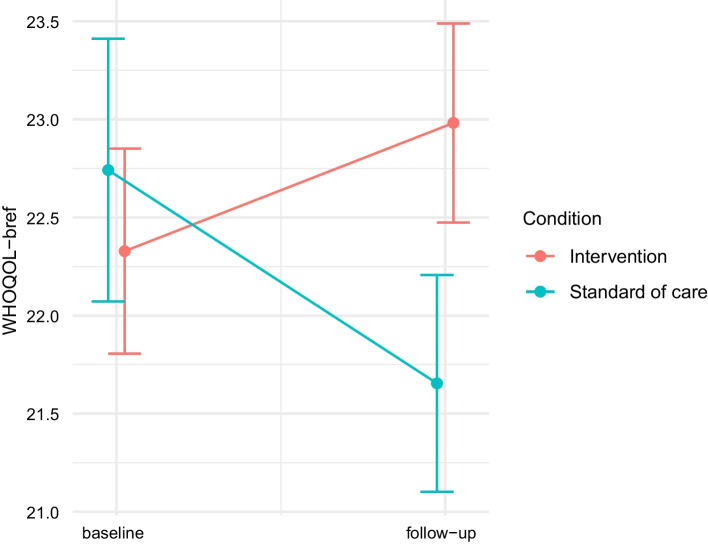
WHOQOL-BREF plot

Firstly, we found a significant main effect of country, indicating higher well-being in Uganda compared to UK. Secondly, we found a significant main effect of Time with a negative coefficient, indicating that participants in the SOC condition reported lower wellbeing at the follow-up time point. We also found a significant and positive interaction coefficient (time × condition), β = 2.22, 95% CI [0.69, 3.76], p = 0.005 (See Fig. 4).

This suggested that the intervention mitigated the decrease in well-being, and possibly even increased it. To assess whether the intervention increased wellbeing in the intervention group relative to baseline, we re-ran the analysis by flipping the dummy variable coding for intervention and control groups. This analysis revealed that wellbeing in the intervention group was significantly increased with respect to baseline: β = 1.16, 95% CI [0.05, 2.27], t(102.42) = 2.07, p = 0.04. Thus, overall, this analysis revealed that whereas the SOC group reported a decrease in wellbeing at follow-up, the intervention group reported increased levels of wellbeing. 

## Discussion

Young people with PAH face challenges to wellbeing, managing the risk of onward HIV transmission and adhering consistently to ART medication. The sharing of one’s HIV status may assist in coping with these challenges and, therefore, supporting young people with PAH with their decisions to share their status should be a priority. The HEADS-UP study is the first study to focus on an intervention for HIV sharing among young adults living with PAH. The study was unique in its inclusion of young people from both high income/low prevalence and low income/high prevalence contexts.

The study was feasible in Uganda with excellent recruitment and retention. High rates of retention have been seen in other longitudinal HIV studies in Uganda [80]. Recruitment and retention were more challenging in the UK. Several UK participants allocated to the intervention condition failed to attend a single session. The difference between the countries may have been due to a variety of factors. Recruitment and retention in the UK appeared to be significantly impacted by the COVID-19 pandemic, which resulted in very limited in-person recruitment (at only one site) and the intervention being delivered remotely. The level of reimbursement could have been more motivating in Uganda than in the UK [81]. Finally, cultural differences may have been important. For example, family members appeared to be more involved in recruitment and in supporting retention in Uganda. There was no evidence of different levels of HIV stigma between UK and Uganda participants.

The intervention was highly acceptable and was perceived to be helpful. Participants identified a range of sharing recipients, consistent with the aim of the intervention. There was minimal evidence of social harms associated with the intervention or with HIV sharing that occurred during the study, although these outcomes did occur. This is to be expected given the inherent difficulty in reflecting on HIV sharing and the risks associated with this behaviour. Nevertheless, sharing that occurred over the course of the study was generally appraised positively by participants.

Unexpectedly, the follow-up support offered was rarely used. This may have been due to this being left to the participants to initiate. It may have been helpful to have provided a specific follow-up individual or group session. The SOC participants rarely discussed HIV sharing in their usual care, which suggests that the intervention is not mirroring already occurring conversations with professionals.

There was tentative evidence that the intervention achieved its aims, from both multivariate (wellbeing, HIV sharing motivation and intention) and descriptive (HIV stigma, hope, HIV disclosure planning specificity) analyses. There was no evidence, however, of an effect of the intervention on HIV disclosure behaviour. This may be explained by the short duration of follow-up (6 months), the rarity of HIV sharing and the fact that we did not select participants due to the availability of a potential sharing recipient (e.g., only including participants with a partner who had not been disclosed to). The latter decision may have reduced the opportunity to detect increases in HIV disclosure. In addition, the measure of HIV disclosure behaviour was of questionable validity. The lack of an effect on HIV disclosure was consistent with some intervention studies [39] but not others [43]. The differences between studies may be due to differences in baseline HIV disclosure motivation, comparison groups, the availability of a potential sharing recipient, and the impact of increasing awareness of U = U resulting in participants feeling that they did not need to share with partners.

There was some evidence of an effect of the intervention on HIV sharing motivation and intention, consistent with a UK camp evaluation of adolescents living with PAH [82]. There was also tentative evidence of an effect on HIV disclosure planning specificity, a construct that has not been assessed in other disclosure interventions. There was no evidence of an effect on disclosure decisional conflict, although the reliability of the DCS was poor. There was a significant effect of the intervention on wellbeing, despite this not being the main aim of the intervention. It is notable that motivational interviewing studies have shown similar findings in relation to psychological outcomes even when the focus is on behaviour change [83]. Aside from the multivariate analysis, the fact that in the intervention group improvements in wellbeing were particularly marked at the post-intervention point, and then waned, suggests an intervention effect. Previous HIV disclosure was related to both disclosure motivation and intention at follow-up. Past disclosure has been to shown to relate to disclosure motivation in other studies [75]. This suggests that past disclosure may have gone well, consistent with the generally positive appraisal of sharing that occurred over the course of the study.

It is difficult to assess the generalisabilty of our findings, particularly given the higher rate of viral suppression than expected [8] and the existence of specialist transition clinics at the recruitment sites. Future studies in sub-Saharan Africa could recruit from district level facilities across different regions. Other factors that complicate interpretation of our findings include the impact of the COVID-19 pandemic, differences in HIV prevalence between countries, different standards of care across sites, and therapist characteristics.

Strengths of the study include the range of outcome variables assessed with good evidence of reliability and validity for most measures (including those that we translated and adapted for use in Uganda). We also made considerable efforts to monitor intervention fidelity. Strengths of the intervention include our efforts to create a programme that was relevant to each study context and allowing participants to choose the type of disclosure recipient that they wanted to develop a plan for. The intervention did not depend on recruiting therapists who were trained mental health professionals, which would have limited its potential for future implementation. In fact, it may have been that the involvement of peer workers, in particular, was crucial to its success.

Limitations of the study include the fact that the comparison condition was not attention matched. In addition, there was no assessment of therapist competence at the end of their training [84]. Attempts to establish the study response rate were not successful with unreliable records of who was approached and refused to participate. It would have been useful to have measured HIV knowledge as there was a specific exercise in session one that aimed to increase confidence in participants’ knowledge about HIV (to assist in HIV sharing). Limitations of the intervention include the need to wait until enough people had enrolled to run therapy groups. Supervision was limited by this being conducted online and, in the case of the Ugandan therapists, not in their first language. In addition, supervision was largely conducted in pairs or with all four therapists together, rather than being individual. Individual supervision may have enabled a safer space for peer workers, in particular, to explore the personal impact of conducting the intervention and providing appropriate support. Regarding the intervention itself, more focus may have been helpful on reflecting on family attitudes to sharing, providing example sharing statements, and outlining external support options, including mental health support. Finally, there were unanswered questions about whether it was appropriate or helpful to include participants with mild learning disabilities.

Future studies could include an adequately powered RCT of the intervention, perhaps comparing the four-session intervention with a single session intervention modelled on session four, along with a cost-effectiveness analysis. A larger sample size would allow for analysis of mediating mechanisms to be undertaken, as well as sub-group analysis (e.g., comparing intervention effects by country). Longer follow-up periods could be used, given the low frequency of HIV sharing. The use of an active comparison group, to control for non-specific therapy factors, would strengthen the design of the study. Future research could develop and test the intervention with younger people with PAH, perhaps soon after HIV naming. In addition, further consideration should be given for how to involve parents and siblings. Many issues associated with sharing an HIV status are similar regardless of the population. Adapting the HEADS-UP intervention for other populations of people living with HIV could, therefore, be undertaken.

The clinical significance of potential intervention effects (e.g., on wellbeing, HIV sharing motivation and intention) is not known. However, the acceptable nature of the intervention, the low frequency of routine discussions about HIV sharing in usual care, and the potential benefits of HIV status sharing suggest that the study might be clinically important. Aside from its efficacy, though, consideration should be given to whether the intervention is implementable. When designing the intervention, we decided that four sessions was the shortest number of sessions to explore the inherent ambivalence associated with HIV sharing in way that was supportive. We continue to believe this is the case, although it may be that the final session can be used as a stand-alone intervention if someone is ready to share. This session took less time to deliver than anticipated.

Regardless of whether the HEADS-UP intervention is delivered in full or in part in routine care, the study suggests that clinics could do more to facilitate discussions about HIV sharing. It may be that developing guidelines and toolkits based on the HEADS-UP and similar interventions would be helpful in this regard. Other suggestions include identifying a professional within each clinic to support HIV sharing, routinely asking patients to identify a person whom they might consider sharing their status with and encouraging the identification of a personal sharing buddy; a person from the individual’s social network who can support decisions to share. The waning of effects between post-intervention and follow-up also suggests that such minimal interventions in routine care might be helpful to maintain the effects of the intervention.

Different ways of delivering the intervention should be considered. We are currently developing and testing a digital, modular, individualised version of the intervention for adults of sub-Saharan African ethnicity living with HIV in the UK. If the group format is retained, one way to reach sufficient people is to carry out the intervention remotely, across countries or even regions. Our study, however, suggests caution given the poor retention in the UK with the remotely delivered intervention. Other options may involve grouping sessions together or tying in sessions with other appointments. The training of therapists (delivered online) was lengthy and considerable ongoing supervision was offered to ensure fidelity to the intervention. This is unlikely to be possible in routine practice. Different ways of delivering training should be considered, for example, online group training and supervision, self-directed and video-supported training. Carrying out the intervention with just peer therapists may be feasible, including young people who have undertaken the programme. This may reduce the training burden. Peer support for HIV sharing has been suggested by young adults living with PAH in a recent study [85].

### Supplementary Information

Below is the link to the electronic supplementary material.

Supplementary file1 (PDF 479 KB)

Supplementary file2 (DOCX 18 KB)

## Data Availability

Anonymised electronic data from the study is available on the Figshare data repository, freely accessible under Creative Commons CC BY licence.
