# Correction: The HIV Empowering Adults’ Decisions to Share: UK/Uganda (HEADSUP) Study—A Randomised Feasibility Trial of an HIV Disclosure Intervention for Young Adults with Perinatally Acquired HIV

**DOI:** 10.1007/s10461-024-04367-2

**Published:** 2024-05-20

**Authors:** Michael Evangeli, Georgina Gnan, Victor Musiime, Sarah Fidle, Janet Seeley, Graham Frize, Annette Uwizera, Matteo Lisi, Caroline Foster

**Affiliations:** 1 Department of Psychology, Royal Holloway University of London, Surrey TW20 0EX, Egham, UK; 2 Makerere University, Kampala, Uganda; 3 Joint Clinical Research Centre, Kampala, Uganda; 4 Department of Infectious Disease, Imperial College London, Imperial College NIHR BRC, London, UK; 5 London School of Hygiene and Tropical Medicine, London, UK; 6 Central and North West London NHS Foundation Trust, London, UK; 7 Imperial College Healthcare NHS Trust, London, UK

In the article, the Acknowledgements text were incorrectly given as “We would like to thank ViiV Healthcare UK, who funded the study, in particular Chris Stainsby and Serufusa Sekidde. We would also like to thank Juliet Ategeka, Jade Robinson and Sam Harris for data entry, Niamh Hinkley for literature searching, and Jane Vosper for therapist supervision. Intervention components were developed with assistance from Bill Miller, Teresa Moyers, Magda Conway, Dorothy Dow, Creative Colony and Bored Digital. We would also like to thank all study collaborators, therapists, members of both advisory groups and all participants” but should have been “ We would like to thank ViiV Healthcare UK, who funded the study, in particular Chris Stainsby and Serufusa Sekidde. We would also like to thank Juliet Ategeka, Jade Robinson and Sam Harris for data entry, Niamh Hinkley for literature searching, and Jane Vosper for therapist supervision. Intervention components were developed with assistance from Bill Miller, Teresa Moyers, Magda Conway, Dorothy Dow, Creative Colony and Bored Digital.

Participating sites were: UK: St. Mary’s Hospital, London: Caroline Foster, Paula Seery, Hana Jayadel, Sara Ayers; St. George’s Hospital, London: Katia Prime, Lisa Hamzah; King’s College Hospital London: Elizabeth Hamlyn, Sally Hawkins; Chiva: Amanda Ely, Abi Carter; Heartlands Hospital, Birmingham: Claire Robertson, Gerry Gillian; North Manchester General Hospital: Katherine Ajdukiewicz. Uganda: Joint Clinical Research Centre: Victor Musiime, Annette Uwizera, William Matovu.

We would also like to thank all therapists, members of both advisory groups and all participants.

The original article has been corrected.

