# Supplementary material for: The HIV Empowering Adults’ Decisions to Share: UK/Uganda (HEADS-UP) Study—A Randomised Feasibility Trial of an HIV Disclosure Intervention for Young Adults with Perinatally Acquired HIV
Source: AIDS Behav. 2024 Mar 15;28(6):1947–64. doi: 10.1007/s10461-024-04294-2 (PMC11161430; doi:10.1007/s10461-024-04294-2)
Supplement: Supplementary file 1 — Supplementary file1 (PDF 479 KB) [file 10461_2024_4294_MOESM1_ESM.pdf]

INTERVENTION MANUAL

UK Version, October 2021

Randomised feasibility trial of an onward HIV disclosure intervention for 18-29 year olds living with perinatally acquired HIV

HEADS-UP

HIV EMPOWERING ADULTS' DECISIONS TO SHARE - UK / UGANDA PROJECT

HEADS-UP Intervention – Session 1

NB: Baseline measures to be completed prior to session 1 by Study Coordinator (SC) – either immediately before, or in the week before, the first session

Materials:

- ***Face to face: stickers, pens, flipchart, marker pens, workbooks, evaluation forms, receipts, Dictaphone, attendance record sheet***
- ***Zoom: electronic version of workbooks (emailed to participants beforehand by SC); Qualtrics link for session evaluation forms (emailed to participants beforehand by SC); blank word documents; attendance record sheet***

Welcome (0-5 minutes)

- Remind participants that they have consented to be being recorded, and that if they miss a session they can ask to see the tape of that session.
- **Start recording – using Dictaphone if face to face, or Zoom recording function (record to Cloud) if using Zoom.**
- Names and role of therapists – write on stickers if face-to-face; zoom name
- Names of participants – write on stickers if face-to-face; if remote, participants to add zoom name (do not need to use actual name)

Ice breaker (5-15 minutes)

Each person in the group gets a turn. When it is your turn, tell the group two things about yourself that are true and one thing that is untrue (this is your lie). Everyone in the group has an opportunity to try to guess which statement was the lie (using the zoom chat function if using zoom). *Once everyone has guessed, the person will reveal what was true and what was false. If only one person was correct, that person can go next*. Let everyone get the chance to go once.

Name and aims of the intervention (15-30 minutes)

Hand out workbooks if face-to-face; ask them to open their workbooks that were emailed in advance if remote (and share screen on workbook).

Each session has some information in your workbook. You can make notes on the workbook if you want to. You can take them with you, or leave them here if you want (if face to face).

If zoom: *There is a link at the front of the workbook to a website that you can use to write on the pdf of the workbook if you don't have a programme that will allow you to do this. Alternatively, you can just take notes as we go along.*

Try not to look ahead in the workbook but, instead, look at the workbook as we go along.

These sessions are about sharing your HIV status. It's normal to think, who should I tell, how much should I share, how will sharing affect me, and similar questions.

The study is called HEADS-UP:

- Explain HEADS-UP acronym (<u>also in workbook</u>)
    - ***H****IV – for people living with HIV from birth*
    - ***E****mpowering – aiming to increase confidence, optimism, and positivity.*
    - ***A****dults – aged 18 to* 29
    - ***D****ecisions – about sharing, under your control*
    - ***S****hare – we are going to use this term rather than 'disclose'.*
        - *Sharing can involve letting someone know who have an illness but not that is HIV or can involve letting someone know you have an illness and that it* ***is*** *HIV.*
        - *Sharing can involve you telling someone yourself, or another person telling someone with or without your permission.*
        - ***Sharing is a process***
            - ***May take place over time – e.g. I have an illness → I have HIV***
            - ***There may be further conversations after sharing***
    - ***U****K/Uganda – both countries at the same time. In the UK, people in London, Birmingham, Manchester and from CHIVA*

- ***P**roject – this is research to see if the intervention is acceptable, feasible (i.e., people will take part) and might help. The aims of the project (also in workbook) are:*
    - *Helping you to make decisions about who to share with, only if you want to*
    - *Helping you to think about how to share your status*
    - *Reducing anxiety about sharing*
    - *Increasing satisfaction with your decisions to share or not to share*

Ground rules (30-40 minutes).

The aim of this exercise is for us to agree a brief list of rules about the group. What do you think the rules of the group should be (write them up on flipchart if face-to-face or type on word document shared screen if remote, and save document)?

If these have not been mentioned by participants, add:

- Confidentiality – not to talk about anyone else outside of the group, including people in the study who are not getting the intervention
- You don't have to share anything about yourself if you don't want to
- There is no pressure to talk in the group if you don't want to
- Not to talk over each other
- No personal criticism of each other. You can criticise ideas, not people
- Attend and arrive/join the meeting on time
- Everyone's opinion counts
- If a session is missed, look at the recording of that session before joining the next session
- Zoom rules and guidance:
    - Do not share meeting link or password with anyone
    - No-one else on screen except you
    - Show yourself at the start of the call so we know it's you and only you
    - No screen recording or pictures
    - Mute when not speaking if noise in the background
    - If you want to speak when someone else is speaking, raise your hand. You can also use the chat function
    - Keep the video on throughout the session
    - Report any concerns about the Zoom session to the therapists

<u>Optional: Participants add list of rules to workbook</u>.

Living with HIV (40-55 minutes)

The aim of this exercise is to encourage accepting, positive and/or helpful beliefs about HIV.

There is evidence that people living with HIV who are more accepting about their HIV not only feel better able to live with HIV, but are also more likely to share.

What are the accepting, positive and/or helpful things that you or other people might say about living with HIV?

Ask the group as a whole (5 minutes) asking not to look at workbook yet

Depending on answers add (state that quotes are from phase 1 interviews) some of:

- Being proud of who you are - "having confidence and knowing that, be proud of who you are you know, because other people have HIV, it's not only you."
- Learning to live with it/it's part of who I am - "I'm happy even if I've got it. Like I can really, I've managed to live with it, I've managed to make it not bother me. I don't even think I've got it sometimes. It's normal to me now, I made it a part of my life. If I could get rid of it then I would but nothing I can do at the moment so I'm just going to accept it."
- Realising it is not a big deal and is ok to share if you want to - "I had like a couple of friends who are out, per se, or like who are just publicly, uhm, people know that they are HIV positive. And kind of like it's a mixture of like being inspired by them and also just realising that sometimes this isn't like a huge deal so I can tell people if I wanted to"
- Being able to shrug off negative comments or attitudes about HIV - "Just seeing things that are positive or other people sharing their experience that helps a lot. But obviously there's always the negative stuff but yeah I don't care because people don't know what it's actually like until they're actually you know in your shoes so. Even when I was in school and we had lessons like sex education you know people always make the jokes about HIV and all this stuff. I mean I never like I just didn't take notice basically."
- I can handle problems related to HIV
- I've learned to live with it

- Dealing with HIV has made me a stronger person
- I've learned a lot because of HIV
- HIV has made life more precious to me
- HIV has made me realise what's important in life
- HIV has taught me to enjoy the moment more
- I am not HIV. I have lots of skills and positive qualities
- People with HIV can achieve the same goals as anyone else
- The only reason why people think negatively about HIV (more than any other chronic illness) is because they've been taught to think this way, not because it's true
- Thoughts are not facts
- I am not alone

Participants to write down suggestions in workbook/therapists to write down suggestions on document, sharing screen - optional)

Learning points

- *Learning to accept HIV before sharing can be helpful*

What do I need to know about HIV to be ready to share (55-65 minutes)?

The aim of this exercise is to think about what you need to know about HIV before sharing.

Without looking at your workbook:

- *What do you think you need to know about HIV to be ready to share, and why?*
- *What do you know about HIV?*
- *What do you want to know about HIV?*
- *What information about HIV do you need to tell someone if you are sharing with them?*

- Depending on answers from group, add some of the following:
    - A healthy looking person can have HIV.
    - HIV is treatable but there is not a cure
    - Coughing and sneezing do not spread HIV.
    - People with HIV can have children who do not have HIV

- There is medication that can be taken before having sex without a condom with someone who is HIV positive that can reduce the chance of HIV infection (PrEP)
- There is medication that can be taken after having sex without a condom with someone who is HIV positive that can reduce the chance of HIV infection (PEP). NB also emergency contraception and STI screening
- If someone with HIV's viral load is undetectable they will not transmit HIV to sexual partners, even when a condom is not used (U=U)
- HIV stands for Human Immunodeficiency Virus and AIDS stands for Acquired Immunodeficiency Syndrome. They are different from each other
- HIV is mainly transmitted by sexual intercourse, or from mother to child (in pregnancy, childbirth or breastfeeding)
- There is a UK law called the Equality Act that protects people with HIV from discrimination.
- In the UK, not sharing your HIV status with a sexual partner is not an offence. It is only an offence if there has been HIV transmission, you know your HIV status, know how HIV is transmitted, have not used a condom and have not disclosed to the partner (reckless transmission).

The above facts are in the workbook, with space to add more

What is important to me? (65-85 minutes)

The aim of this exercise is to reflect on personal values, and to think about how HIV sharing fits with personal values

Therapists asks the group, "*What are personal values?*"

Depending on responses, add the following (in workbook) (5 minutes):

- A trait or quality which a person considers worthwhile and important
- A direction you want your life to head in
- Different from goals, which are stepping stones along the way. For example, a value might be wisdom, and the goals in line with this value might be to gain an education or to ask advice from others.

I now want you to look at this list of personal values in your workbook (on Zoom, share screen on workbook with names of values on them). *Your task, **on your own**, is to rate which*

ones that are not important to you (NI), quite important (QI), and very important to you (VI). Then, from the 'very important' list, choose the 3 most important personal values for you. Finally write down the 3 most important personal values in your workbook <u>(space in workbook to add personal values)</u> (10 minutes). *If you need any help, please ask us.*

List of values:

		NI	QI	VI
Acceptance	To be open to and allowing of things I dislike or I don't want in myself, others, and the world around me			
Adventure	To be adventurous; to actively explore new and challenging experiences			
Assertiveness	To stand up for my rights and request what I want, whilst being respectful both to myself and others			
Authenticity	To be authentic, genuine, and real: to be true to myself			
Caring/Self-Care	To take care of, or act caringly toward myself, others, the environment and all living things			
Compassion	To act kindly toward myself and/or others when in pain			
Connection	To engage fully in whatever I'm doing; to give the activity, or the person I'm with, my full attention			
Contribution	To contribute, give, help, assist, share or be generous			
Cooperation	To be cooperative and collaborative with others			
Courage	To be courageous or brave; to persist in the face of fear, uncertainty, and threatening circumstances			
Creativity	To be creative or innovative, at work or at play			
Curiosity	To be curious, open-minded and interested; eager to explore, discover and learn			
Encouragement	To encourage and reward behavior that I value in myself or others			
Excitement	To seek out, create or engage in activities that are exciting, novel or stimulating			
Fairness/Justice	To treat myself and others justly and fairly; to stand up for fairness and justice			
Fitness	To maintain, improve, or look after my physical and mental health			
Flexibility	To adjust and adapt readily to difficult or changing circumstances			

Forgiveness	To be forgiving toward myself or others; to let go of blame and resentment			
Freedom	To live my life the way I want to, and help others to do likewise			
Friendliness	To be warm, friendly, open, accepting, agreeable, and helpful toward others			
Fun	To be fun-loving, playful, light-hearted, humorous; to seek, create, encourage, or engage in fun-filled or humorous activities			
Gratitude	To be grateful for and appreciative of myself, others, and the world around me			
Honesty	To be honest, truthful, and sincere with myself and others			
Industry	To be industrious, hardworking, committed, proactive or dedicated			
Intimacy	To open up, reveal, and share myself with others – emotionally or physically or both			
Kindness	To be kind, considerate, nurturing, or caring toward myself, others, or the world around me			
Love	To act lovingly or affectionately toward myself or others			
Mindfulness	To be mindful; open to, engaged in and curious about your here-and-now experiences			
Order	To be orderly and organised			
Persistence	To continue resolutely with your task or challenge, despite problems, fears and difficulties			
Respect	To treat myself and others with genuine care and consideration			
Responsibility	To act responsibly and be accountable for what I do			
Safety	To secure, protect or ensure my own safety or that of others			
Sensuality	To seek, create or enjoy pleasurable sensual experiences			
Sexuality	To explore, express, or assert my sexuality			
Skillfulness	To continually practice and improve my skills, and apply myself fully when using them			
Supportiveness	To be supportive, helpful and available to myself or others			
Trust	To be trustworthy; loyal, faithful, sincere, and reliable; and/or be trusting of others			
Other Values	Other personal qualities you want to embody? Other ways you want to treat yourself, others, or the world around you?			

Elicit responses about exercise (5 minutes)

In the final session we will ask how your sharing goals relate to these personal values

NB If running out of time ask participants to do this exercise for homework

Summary (85-90 minutes)

- Praise everyone for contributing
- Ask group for main learning points verbally
- Expenses if travel to face to face session
- Mention post session evaluation form (hand out if face-to-face; Qualtrics link emailed by SC if remote), stating that it should only take one or two minutes to complete.
- Give contact details of peer worker if participants want to contact between sessions
- **Provide details of next session**
- Tell participants that if they are unable to make a session time, there will be the opportunity to review a recording of the missed session.
- **Stop recording**
- Record attendance

HEADS-UP Intervention – Session 2

Materials:

- ***Face to face: Flip chart, pens, marker pens, A4 quiz cards, stickers, computer/data projector/screen, evaluation forms, receipts; link for film; Dictaphone; attendance sheet***
- ***Zoom: HIV sharing quiz questions in a document; Qualtrics link for evaluation; link for film; HIV sharing quiz questions and answers documents; attendance sheet; blank word documents***

Start recording – using Dictaphone if face to face, or Zoom recording function (record to Cloud) if using Zoom.

Recap and Review (0-5 minutes)

- Any thoughts or questions from last time, reminding the participants about the content of session 1

HIV sharing quiz (5-40 minutes)

The aim of this exercise is to increase your knowledge of possible causes and consequences of sharing

Questions printed on an A4-sized card if face-to-face or on document to be shared if Zoom.

Read out the first question and then say:

Without looking at your workbook, do you think the answer is true or false, and why do you think the answer is true or false?

Allow the group to relate the question to their own experience

Who would like to read out the next question? Give card to that person or ask them to read out a question if Zoom. The participant reads out the question.

Without looking at your workbook, do you think the answer is true or false, and why do you think the answer is true or false?

Allow the group to relate the question to their own experience. Repeat until all 10 questions are asked. Then, at the end, go through the questions and answers verbally if face to face, or using shared screen with question and answer document if on Zoom.

1. It's very unusual to be worried about sharing your status
   False – anxiety about sharing is very normal. This doesn't mean that sharing will go badly

2. Most people share selectively e.g. they don't share with everyone they know

True – most people tell someone but not everyone

3. People are less likely to share with their partner if they are in a regular compared to a casual relationship
   False – this might be due to greater feelings of trust and more relationship commitment in a regular relationship

4. If you share your status you are more likely to receive social support from the person you share with
   True. This can be emotional support or practical support.

5. People's reactions to you telling them that you are living with HIV cannot change from negative to positive over time
   False - Reactions can change from negative to positive over time

6. It is common for young people born with HIV to share their status with few people
   True – although other people may have been told by family members

7. Sharing can increase confidence in living with HIV
   True – this could be because of the experience of sharing itself, or because of good responses from others

8. Some people experience a negative response from people when they share (T)
   True – some people are not supportive

9. Most people respond well when you tell them (T)
   True. People can be supportive, caring and understanding. They may feel good about being trusted enough to be told

10. Sharing your status can help you to take your medication everyday (T)
    True. Sharing is linked with better adherence but it's also possible to adhere well without sharing

Elicit views of the group and summarise reasons to share and not to share (which can be personal - internal reasons – or social – reasons involving other people). Consider adding some reasons from the list below.

What reasons are there for not sharing an HIV status?

- REASONS NOT TO SHARE
  - PERSONAL
    - Not having to:
      - Due to U=U
      - As status doesn't define me
      - Don't owe it to anyone
      - HIV not being a big issue, so doesn't need to be shared

        - Not needing support
        - Not minding it being a secret
    - Negative attitudes about HIV and HIV sharing
        - Due to ethnicity
        - Due to religion/Attitudes of church
        - Being told by mum/step-parents not to share/ Family secrets and family not having coming to terms with it/not being prepared by family
        - Only medication spoken about in my culture, not sharing
        - Self-stigma re HIV; Not having embraced HIV+ status; Being embarrassed about status. Thinking HIV is a punishment/difficult to accept HIV and integrate it with my view of myself
        - Talking about it makes me feel different
        - Talking about it makes me feel bad
        - Finding it uncomfortable to talk about
    - Not knowing enough about HIV
    - Not feeling that keeping diagnosis to myself feels like keeping something from people
    - Not feeling confident to/not having the courage to
    - Not being ready
    - Used to not sharing (becomes a habit)
    - Personality – being a private person
    - Other fears
        - Fear of losing control
        - Fear of feeling different/feeling alone
        - "It can ruin lives"
- SOCIAL
    - Fear of how other people will respond:
        - Being talked about/they will tell others (including on social media)/trust will be betrayed (now or in the future)
        - Being bullied
        - Being isolated
        - Not knowing how people will react
        - Not wanting to be looked at differently
        - Being pitied
        - Losing friends or partner
    - Protecting others
        - Fear of outing parents
        - To protect others
        - Not wanting to distress others
        - Not wanting others to be uncomfortable
        - Not wanting to burden others
    - Other thoughts about the other person
        - Partner not being potentially long-term

- Not knowing/trusting the person
- Thinking that others don't know about U=U

What reasons are there for sharing an HIV status?

- REASONS TO SHARE
  - PERSONAL
    - Reducing negative feelings
      - Feeling unwell/being sick
      - Feeling exhausted lying
      - Feeling claustrophobic not telling
      - Wanting to stop feeling bitter
      - Not wanting to deceive the other person
      - Feeling guilty about not having shared
      - Feeling burdened by not sharing; to get it off your chest; to unburden
      - Not wanting there to be barriers or secrets between me and others
      - Not wanting to keep quiet anymore/thinking 'what's the point of keeping quiet'
      - Anticipated regret – not wanting others to ask 'why didn't you tell me'; infecting others; a person hearing it from others; Keeping this a secret might make people wonder what else I been keeping from them (once they find out); Not wanting a partner to find out too late - not wanting to break their trust
    - Hoping for positive outcomes for self
      - Feeling catharsis
      - Wanting to show true self
      - To feel less alone
      - Sharing to normalise/feeling normal is important
      - Not wanting to keep secrets/lie anymore/valuing honesty/wanting to be free and open
      - Thinking that sharing lowers self-stigma
    - Feeling good, thinking positively and resiliently
      - Seeing sharing experiences as learning experiences rather than focusing on any negative reactions
      - Knowing people all react differently and being prepared for that/feeling ready for any outcome
      - Knowing there are others with HIV
      - Feeling supported and not alone
      - Meeting others with HIV
      - Naturally wanting to share
      - Not being affected by what others think
      - Blaming others for their negative reactions rather than myself and the diagnosis

        - Need to be OK with having HIV to share
        - Feeling good in myself
        - Being ready for the outcome/thinking other people are entitled to feel angry
        - Thinking that it's easier for others to deal with it if you're born with it
        - Feeling healthy and looking good
        - Feeling comfortable with the idea of people knowing
        - Feeling self-confident; having a confident personality; determination; feeling confident
        - Understanding HIV; knowing U=U
        - Previous positive experience of sharing
        - Family openness re HIV
        - Being brave
        - Wanting to prove people wrong – I have potential, I'm no different, I can still achieve my dreams
        - Experiencing how it feels to be open about HIV status at CHIVA camp
        - Having the attitude that if someone has a negative reaction you are better off not being their friend anyway
        - Realising that people can only put you down if you let them affect you
        - Expecting that there may be a negative reaction but being prepared to deal with this
    - Moral beliefs and values
        - Feeling like the right thing to do
        - Feeling passionate about HIV
        - Thinking that if I was in their situation, I would want to know
        - Having a personal rule about telling partners
        - Feelings of responsibility to others/owing others the truth
- SOCIAL
    - Educational
        - To educate others and to reduce stigma;
        - Want to show people a success story
        - Giving hope to other people and showing them that having HIV is not the end of the world/helping others with HIV
        - Sharing to explain why taking medication
    - For benefits to self, including support
        - To get help in the future
        - For validation
    - To strengthen/assess nature of relationships or because of the nature of the relationship
        - To test friendships
        - Wanting no barriers in a relationship/to get close to people
        - Sharing due to comfort in relationship;

    - To deepen the relationship
    - Relationship getting serious and wanting to make a commitment
    - Having had sex
    - No-one else to tell
- Positive beliefs about the other
    - Trusting others/thinking they would react OK
    - Telling people as a reward for loyalty
    - Having good friends who care about you
    - Feeling close to the other person
    - Sharing easier if someone has similar experiences, e.g., loss or LT illness
- Influence of others' behaviour
    - Being asked
    - If friend tells you something about themselves/If someone shares something with you, you feel safer to share with them
    - Seeing other people sharing
    - Positive reactions from the first people you share with help you gain confidence to share with others
    - Thinking people know already
- Harder to hide meds from partner or others and not being on top of medication

Learning points:

- *There is often a mixture of reasons to share or not to share (pros and cons). These reasons might be different depending on who we are thinking of sharing with*
- *It's rare that you will <u>need</u> to share. Secrets are part of everyone's life – smoking, sexuality, failing at something. There may be some situations where it is best not to share, e.g. for safety, and other situations when it is best to share*
- *How you feel about HIV may affect your decisions to share*
- *Telling someone is completely up to you*

Anxiety about sharing (40-50 minutes) – in the workbook

The aim of this exercise is to think about helpful things to think and to do to manage anxiety about sharing.

Feeling anxious about sharing your status is <u>normal</u> but there might be helpful ways of thinking and behaving to manage this anxiety.

Exercise: Ask group – *what are helpful things TO DO and TO THINK/SAY TO MYSELF in managing anxiety about sharing?* (Optional prompt – *What helps you when you feel anxious at other times in your life?*)

Optional - Write answers on flipcharts if face-to-face or on document using shared screen if remote (and save) – TO DO and TO THINK/SAY TO MYSELF.

Depending on answers, add:

TO DO
- Find your support network (including healthcare workers) of people who will understand. Talk to them about sharing. Use them to help you share/ask them to share for you/talk to them after sharing. Learn from others' experiences of sharing.
- Talk about HIV at home
- Building sharing skills
- Building up slowly (e.g., talking about HIV in general before sharing)

TO THINK/SAY TO MYSELF
- Be kind, encouraging and non-judgemental to myself
- Feel confident about myself
- It's a challenge I can overcome
- Am I right to be so concerned about this?
- What is the evidence that what I'm worried about is going to happen?
- Could I live with it if what I fear happens?
- What would I say to someone I cared about who was in this situation and had this concern?
- What is the most likely outcome of sharing?

Ask participants to write down responses in workbook

Summarise responses

Video 1 - Experiences of sharing – Film is 7m46s (50-65 minutes)

The aim of this exercise is to reflect on the sharing experiences of a young person living with HIV

I'm going to play a video of someone living with HIV talking about their experiences of sharing their status. This person has agreed to be filmed but it's important not to talk about her or the video outside of the sessions as she has only agreed to do this for these sessions.

- Play film (using shared screen if Zoom). Tick 'Optimize for full-screen video clip' to show film. Untick this option after film is shown.
- Questions
    - *How did she approach sharing?*
    - *What type of responses did she receive from others?*
    - *How did she deal with challenges?*
    - *How was her story similar or different to yours?*

Personal guidelines about sharing (65-85 minutes)

Whole group exercise.

Optional - Write down answers on flipchart if face-to-face; on word doc on shared screen if remote (and save)

The aim of this exercise is to think about personal sharing guidelines. Having personal rules or guidelines can be helpful in different areas of life (for example, "I will only drink alcohol on a Friday or Saturday").

*Regarding sharing, it can be difficult to know who to tell and when? Maybe having some **personal** guidelines about sharing can be helpful.*

For example,

- *I'll share if I'm serious about someone*
- *I'll share if I'm comfortable with a partner*
- *I'll tell the most important people in my life*

1. *What benefits can you think of having personal sharing guidelines* (ask the group and reinforce responses, e.g., affirm)?

Depending on responses add:

- May increase confidence about making better sharing decisions
- May help in planning sharing
- May help in guiding when to share and who to share with
- Acknowledge that there might be disadvantages of personal guidelines, e.g., too many exceptions

2. *When do you think that sharing your status with someone else would be the right thing to do?*

Reframe responses in the form of personal sharing guidelines.

3. *When would it not be the right thing to do?*

Reframe responses in the form of personal sharing guidelines.

Optional - Write down elicited guidelines on flipchart if face-to-face, or on word doc using share screen if remote (and save)

Learning points:

There may be different guidelines for different people. Any guidelines are personal rather than being something that everyone/the group needs to agree to. Also, guidelines don't need

to be followed every time – there may be reasons to do something different in particular situations. They are your guidelines and you can break them!

Summary (85-90 minutes)
Praise everyone for contributing
Ask group for main learning points verbally
Expenses if face to face
Post session evaluation form (handout if face-to-face; Qualtrics link sent by SC if Zoom)
Provide details of next session
Stop recording
Record attendance

HEADS-UP Intervention – Session 3

Materials:

- ***Face to face - Flip chart, pens, marker pens, stickers, computer/data projector/screen, evaluation forms, receipts, vignettes on laminated cards, Dictaphone, film, attendance sheet***
- ***Zoom – Jamie and Becky Film, link for evaluation forms, vignette document, attendance sheet, blank word documents***

Start recording – using Dictaphone if face to face, or Zoom recording function (record to Cloud) if using Zoom.

Recap and Review (0-5 minutes)
Any thoughts from last session, reminding the participants about the content of session 2

Video 2 - Jamie and Becky (5-25 minutes, NB film is 7m40s)
The aim of this exercise is to think about possible ways that HIV can be shared

*We are now going to show you a film showing someone sharing their HIV status to a partner. It's important to say that it's a **fictional** account just showing **one way** that HIV sharing might take place. Some parts of the film might be difficult to watch and it might remind of something in your own life. This is really normal and if anyone feels upset when they are watching it, or afterwards, **please** make contact with us so that we can offer support. You can use the zoom chat function to privately message us if you want. Try to watch the film carefully, as we'll ask you some questions about it straight afterwards.*

If remote, show film by sharing screen. Tick 'Optimize for full-screen video clip' to show film. Untick this option after film is shown.

After the film, ask the group some (e.g., up to 3) of the following questions:

- *What did you think of the way that Jamie shared his status?*
- *How would you have felt if you were Jamie?*
- *How would you have felt if you were Becky?*
- *What did Jamie do well/what worked well?*
- *What could he have done differently?*
- *What would you have done differently?*
- *Which parts of the film were most realistic?*
- *How different is the situation in the film from sharing with a friend?*

Summarise responses.

<u>Learning points</u>

- *Anxiety about sharing is really normal but often the anxiety will be worse than the reality*
- *Trying to provide clear information about HIV can be helpful*

- *There may be a mixture of responses from the person you share your status with, some of which change over time.*

If you've decided to share to a particular person (25-45 minutes)

The aim of this exercise is think about where, when and how to share, and what to say

If you have made a decision to share with a particular person, what do you think you need to consider about where, when, how to share and what to say? (ask group as a whole).

Optional - Elicit responses and add to with some of the points below on separate flip chart sheets (if face-to-face; if remote, write on word doc while sharing screen, and save) (WHEN, WHERE, HOW, WHAT TO SAY)

WHEN
- When you're relaxed – not when either of you is stressed or busy – like around exams or with a big event coming up. You need to feel calm and confident so you can deal with however they react.
- In a relationship:
    - Not too early (before you trust them)?
    - Not too late (when they might say why didn't you tell me before/why didn't you trust me?).
    - Not too aroused, e.g., just before or during sex

WHERE
- Do it somewhere where the person can leave if face to face
- Face to face, don't do it over the phone?
- In a letter?
- Safe place - in public, checking in with someone else, check there is a way to escape.
- Pick a place where you feel comfortable to talk openly, without disruption. It could be place that's special for you (so, if you have grown up together, a place which has good memories), or somewhere where you will both be relaxed and comfortable, or at home.
- At clinic appointment? If your doctor helps you share it might seem more serious but they can explain and answer any questions

HOW
- Keep calm
- Say it in a matter-of-fact way
- Take your time and give the other person time
- Be yourself
- Be honest
- Be simple and clear
- Good eye contact
- Confident body posture

WHAT TO SAY

- Talk with them in general about HIV. You could start up a conversation about something you read or saw on the TV or online or ask them how they would react if they tested positive. See what they say and how they react.
- Consider what you want to tell this person and how they may react. Think about what they may be anxious about if they knew you were living with HIV, and what information you could give them.
- Think back to when you were told yourself– or how you would like to have been told. This should help you think about the best way to say what you want to say.
- Start off gently, e.g., “There’s something I want to tell you that I’ve wanted to say for a long time.”
- Learn a way to tell your story in a personally meaningful way – use ‘me’, ‘my’, ‘I’ and ‘HIV’; be specific
- Explaining to the person how you feel and why you feel this way/why you have decided to share your status (e.g., you care about them, you trust them).
- Think about whether you want to tell them how you became HIV positive – you may not want people to know about the HIV status of your mother. That’s fine – you don’t need to say that you were born with HIV and you may not want people to feel sorry for you. On the other hand, you may want people to know that you are not living with HIV because of anything that you did.
- Think about how you’re going to explain to them that they need to keep this information private. Explain that this is because not everybody is understanding or has the right information about HIV.
- Provide information on HIV. For example:
    - I live with HIV. It is a virus that makes my immune system weak so I cannot fight off infections easily like other people without HIV. So I take medication which makes my immune system strong. HIV doesn’t stop me from doing everyday activities, like sports, swimming, etc.
    - Be prepared with basic information about HIV - transmission; how treatment works; prognosis; having children; testing; U=U; relationships; work and everyday activities. (The ‘Young People’ section on www.chiva.org.uk and NHS websites are really helpful to remind you about the HIV facts)
    - The right words to use
- Encourage any questions - Let them know they can ask you anything now or in the future.
- Explain what you would like from them (e.g., to make their own decision about whether to stay with them)
- Think about suggesting they come with you to clinic

Summarise responses and ask participants to add to their workbook (either printed out version or electronic version)

Learning points to add:

- *Having a plan about when, where, how and what to say, may be helpful*

- *Talking the plan through with someone you trust might be helpful, as well as practicing in your mind or with someone.*

Practicing sharing (45-70 minutes)

The aim of this exercise is to gain experience in sharing and in dealing with different responses; to practice a way of sharing that you feel comfortable with.

- Ask group to look at vignettes on laminated card (one card each) if face to face, or if using zoom, share screen with vignettes document (or ask to look at vignettes in workbook)
- Vignette 1 – you are living with HIV. You have known your best friend for many years but you have not told them about your HIV status. You trust them and feel comfortable with them.
- Vignette 2 – you are living with HIV. You have started seeing a new partner and it seems as though the relationship is getting serious. You have not had sex with them yet but you would like to.

Choose one of the two vignettes. One person plays the character living with HIV and shares their HIV status with the other person. The second person can react however they like but don't tell the other person how you're going to respond beforehand. It's OK to express some negative emotions (e.g., anxiety, low levels of anger). The first person responds.

- *Try to:*
  - *Respect the rights of yourself and the other person*
  - *Show good eye contact, and a confident body posture*
  - *Say what I feel, why I feel this way and what I'd like, clearly and honestly*
  - *Think about some of the suggestions from the last exercise*
- *Switch around after 5 minutes so that both of you have a turn playing the person living with HIV*
- *In pairs (if using zoom, use Zoom Breakout Rooms function https://www.youtube.com/watch?v=jbPpdyn16sY)*
- *Don't worry about whether what you're saying is right or wrong. Just try things out and see how they feel*
- *If you would like us to join your breakout room to observe the role play, please let us know*

If any participant does not want to do the role play, they can choose to write down what they would say and share this with their partner, just play the role of the person being told, or can watch others.

Feedback

- *What went well? What went less well?*

After sharing (70-80 minutes)

You may get no reaction, a positive reaction or a negative reaction from the person you tell. They may say:

- *How did you get it?*
- *How am I sure you have not infected me?*
- *Will you fall sick any time soon?*
- *Why didn't you tell me before?*
- *You don't seem unwell to me*

What do you think would be helpful TO THINK and what would be helpful TO DO after you share if you get response like this (for example, the other person saying "Why didn't you tell me before?") (ask group as a whole).

Optional - Elicit responses and add to with some of the points below on a flip chart sheet (if face-to-face; if remote write on word doc while sharing screen) (TO THINK and TO DO)

TO THINK

- If someone does not respond as well as you would have liked, it might be helpful to think:
    - It says more about them than it does about me.
    - Don't let a negative reaction change me
    - Whatever their reaction may be at first, these can change in time and with more education about HIV
    - It does not mean that I won't have positive reactions in the future.
    - Try not to take their response personally
    - Accepting the outcome whatever it is.
    - Life goes on
    - It's their loss because they've lost out on a friend/a relationship
    - Being rejected is the easiest way to remove unwanted people from my life (who are not worthy of me)
    - If you can't take this information then you're obviously not meant to be in my life
    - Their response may be due to them:
        - feeling afraid about getting HIV, needing to test, the unknown, or you becoming unwell
        - feeling angry or confused or hurt about you putting them at risk or you not telling them before
        - feeling surprised or shocked
        - feeling concerned about what other people will say
        - wanting to show you that they can deal with it
        - being afraid that you are going to die
        - wanting to protect you
        - needing to be reassured
        - not being honest with you about how it makes them feel
    - Don't let their questions offend me. They're just trying to understand.
    - Even if they react badly, they probably just need a bit of time to think it all through.

TO DO

- If they say something incorrect about HIV, politely correct them and suggest they look at helpful websites
- Revisit the conversation later and check in about how they are feeling
- Continuing to talk about HIV, stating how you feel, why you feel this way and what you would like
- Ask for support from the other person
- Provide support for the other person
- Offer an opportunity for them to speak to your doctor?
- Keep calm
- Keep safe
- Seek support – friends, family, partner, church?
- If they ask you a question you cannot answer, just say, I am not sure about that, but I will get back to you with the correct information. Don't try and answer or explain anything you are not sure about.
- If condom came off or broke, or you didn't use one – it's not too late. PEP, emergency contraception and STI screening.
- Think about whether anyone else might need support, e.g., mother.

Elicit response and ask participants to add to workbook

Information on next session and follow-up period (80 - 85 minutes)
- *The next session will be an individual session to focus on your sharing goals.*
- *After that session, we will be offering:*
    - *Peer worker support for you and anyone you share your status with over the 6 months from the start of the sessions. We'll provide more information on this next session*
- *If you want to keep in contact with each other that's fine* (suggesting using chat function on Zoom to swap numbers – to everyone or privately). *You might want to just have a Whatsapp group, or to meet together online, or even in person.*

Summary (85-90 minutes)
Praise everyone for contributing
Ask group for main learning points
Expenses if face to face
Post session evaluation form (or SC to send Qualtrics link if remote)
Provide details of next session
Stop recording
Record attendance

HEADS-UP Intervention – Session 4

Materials:

- ***Face to face: pens, workbooks; sharing plan proforma, session evaluation questionnaire, receipts, Dictaphone, attendance sheet***
- ***Zoom: sharing plan proforma document; Qualtrics link for session evaluation questionnaire, attendance sheet***

Start recording – using Dictaphone if face to face, or Zoom recording function (record to Cloud) if using Zoom.

Recap/Review (0-5 minutes)

- Any thoughts from last session, reminding the participant about the content of session 3

Aim of the session (5-10 minutes)

This individual session aims to help you develop a sharing goal and plan of how to achieve this goal using a Sharing Proforma

You will see in your workbook that there is a sheet (repeated 3 times) where you can write down a sharing goal and a plan about how to achieve that goal. I am going to try to help you to fill out one sheet.

Assessing Sharing Goals (10-35 minutes)

- **What is my sharing goal?**

A sharing goal might be something like this: to share with my brother, to think about sharing to my partner, to think about who to share with, to decide whether I want to share with my friend/anyone, to share with my next partner

Use proforma (in workbook or on document using shared screen if using Zoom) to try to generate a sharing goal.

If participant is struggling, move on to values and personal sharing guidelines

If you can't think of a sharing goal, can you remember what the 3 personal values were that you chose in session1?

Can you remember what your personal sharing guidelines were from session 2?

Then ask the following questions to try to elicit a sharing goal:

- *And how do those ideas apply to your own life?*

  - *Do those sound like the right guidelines for you when deciding whether to share your HIV status with someone else?*
  - *How might these ideas fit with a sharing goal?*

- **Why is this my sharing goal?**

This could be something like: I want to tell the truth; I want to feel less alone; I think they will react well because I have a good relationship with them; I want to make the right decision; I want to be honest with them

Use proforma to try to generate reasons. Also can use list of reasons to share from session 2 and/or ask:

  - *What would life be like/the best thing be if you were able to achieve this goal?*
  - *What would concern you about not achieving this goal?*
  - *What would the benefits be of achieving this goal?*

- **How does this goal relate to my personal values and personal sharing guidelines? What are my personal values? What are my sharing guidelines? Why is this reason for sharing important to me?**

Personal values *could be those you wrote down in session 1, for example, wanting to be honest.* ***Personal sharing guidelines*** *could be something like: I'll share if I'm serious about someone; I'll share if I'm comfortable with a partner; I'll tell the most important people in my life*

Use proforma to help participant to focus on personal values and personal sharing guidelines. Also, can ask:

  - *What is most important to you/what is most important about the way you want to live your life?*

Ask participant to write down the answers to these questions in their workbook.

Developing an Action Plan (35-60 minutes)

Now we're going to work on a plan about how to achieve the goal.

When to share?

This could be something like: before we have sex for the first time; at a particular time of day.

Use proforma to generate response. Refer to session 3.

Where to share?

This could be something like: a safe place, face to face

Use proforma to generate response. Refer to session 3.

How to share and what to say?

For example: what to say – how I feel, why I feel that way; what I'd like; starting off gently; explain why I've decided to share; giving information about HIV; stressing importance of them not telling others; how you are going to say it, e.g., keep calm

Use proforma to generate response. Refer to session 3.

OR, IF PARTICIPANT IS STRUGGLING TO DEVELOP A PLAN

What needs to change for you to share? What will help you feel safe to share? What will help you to decide whether to share?

For example: If I felt that I had to; If I trusted that person more; Feeling more confident about saying the right words; being less worried about how they would respond; finding someone to support me – someone with HIV who has shared; listing the pros and cons of sharing

Use proforma to generate response. Refer to session 3. Also consider asking:

- *What's stopping you from sharing right now?*

Ask participant to write down the answers to these questions in their workbook.

Developing a Coping (if...then) Plan (60-80 minutes)

Now we're going to think about barriers to sharing.

What could be a barrier to sharing taking place, or to sharing going well? What could be a reason not to share?

For example: they don't respond well; I don't say everything I had planned to say; I'm worried it will change the relationship; I'm worried they will reject me; feeling anxious; my mum doesn't want me to share

Use proforma to generate response. Also refer to reasons not to share from session 2 and/or consider asking:

- *How likely is it that that you will achieve the sharing goal?*

<u>Try to frame the barriers in terms of anxiety</u>, then ask participant to provide more detail to understand what they are particularly anxious about.

What could I do or say to myself to manage this barrier/these barriers?

For example: talk to someone I trust; sharing this plan with someone else; say to myself that they might feel differently after time; try not to take it personally; accepting the outcome;

saying that they're just trying to understand; keeping calm; talking to mum first; asking for support in talking to mum

Use proforma to generate response. See sessions 2 (Sharing Anxiety Model) and 3. Also consider asking:

- If barriers are related to motivation, *ask yourself*:
    - *Am I right to be so concerned about this* (e.g., friend reacting badly; mum being angry that I shared)*?*
    - *What is the evidence that what I'm worried about is going to happen? For example, if you are worried that the person you are going to tell is going to tell others:*
        - *Can they keep private information private and not gossip about what you tell them? Have you tried telling them another secret? Did they keep it? If you haven't tried, you can try now and see if it gets out or if you can trust them.*
    - *Could I live with it if what I fear happens?*
    - *What would I say to someone I cared about who was in this situation and had this concern?*
    - *What is the most likely outcome of sharing?*
    - *Would it be helpful to just focus on what I can control?*
- If barriers are related to confidence:
    - See session 3 and consider role playing
    - Ask, *Have there been situations in the past where you have felt anxious about sharing but have shared and things have worked out well?*

Ask participant to write down the answers to these questions in their workbook.

Ask the participant:

Are you still happy with your goal and plan, or do you want to change this?

Checking that the plan is specific, measureable, achievable, relevant and time-bound (SMART)

Discuss possible benefits of role playing or rehearsing the plan

Summary (80-90 minutes)

- Elicit feelings about and learning points from the session and intervention as a whole and any questions, Provide affirmation and respond to questions, Elicit response to this.
- Expenses if face to face
- Session evaluation questionnaire, or SC to provide Qualtrics link for session evaluation questionnaire if using Zoom.
- Post-intervention support – give details of peer worker for self and disclosure recipients; Call to leave message; text; Whatsapp; online information. NB Manage expectations about availability of peer worker. Not for emergencies. May take up to 2 weeks to respond. Provide details of other support options.

- Reminder about 6 month measures (i.e., in approximately 5 months' time) and sub-study interview if chosen.
- **Stop recording**
- Record attendance
- Record whom the sharing recipient was (on Session 4 proforma – with example)

Post-intervention measures to be filled in immediately after session 4 or plan for filling in within next week

- NB Reimbursement electronically by SC after measures filled in

Sharing Proforma

Sharing Goal

What is my sharing goal?
(e.g., to share with my brother, to think about sharing to my partner, to think about who to share with, to decide whether I want to share with my friend/anyone, to share with my next partner…)

………
………
……..

Why is this my sharing goal? What are the reasons behind this goal?
(e.g., I want to tell the truth; I want to feel less alone; I think they will react well because I have a good relationship with them; I want to make the right decision; I want to be honest with them)

……..
……..
……..

How does this goal relate to my personal values and personal sharing guidelines?
*(**personal values**: e.g., wanting to be honest; **personal sharing guidelines:** e.g., I'll share if I'm serious about someone; I'll share if I'm comfortable with a partner; I'll tell the most important people in my life)*

………
………
……..

Action Plan

When to share?
(e.g., before we have sex for the first time; at a particular time of day)

………
………
……..

Where to share?
(e.g., a safe place, face to face)

……
……
……

How to share and what to say?
(e.g., what to say – how I feel, why I feel that way; what I'd like; starting off gently; explain why I've decided to share; giving information about HIV; stressing importance of them not telling others; how you are going to say it, e.g., keep calm)

……
……
……

OR, IF DEVELOPING A PLAN IS DIFFICULT

What needs to change for you to share? What will help you feel safe to share? What will help you to decide whether to share?
(e.g., If I felt that I had to; If I trusted that person more; Feeling more confident about saying the right words; being less worried about how they would respond; finding someone to support me – someone with HIV who has shared; listing the pros and cons of sharing)

……
……
……

<u>Coping (if….then) plan</u>
What could be a barrier to sharing taking place, or to sharing going well? What could be a reason not to share?
(e.g., they don't respond well; I don't say everything I had planned to say; I'm worried it will change the relationship; I'm worried they will reject me; feeling anxious)

……
……
……

What could I do or say to myself to manage this barrier/these barriers?
(e.g., talk to someone I trust; sharing this plan with someone else; say to myself that they might feel differently after time; try not to take it personally; accepting the outcome; saying that they're just trying to understand; keeping calm)

……
……
……

Peer worker intervention follow-up support

Materials

- Attendance sheet

Objective:

To provide follow-up ***emotional, informational and problem-solving support*** (in the 6 months from baseline to the follow-up data point) from a peer worker for both young people in the intervention condition and people whom they have shared their status with.

Strategy/procedure:

- Follow-up support will involve the use of both phone and online/social media options.
- Participants will be given a phone number and email address to contact the peer worker on.
- Hours of phone support availability will be clearly stated (e.g. 2 half days a week, 1-2 hours on different days of the week, one full day), and online information (permanently available) will be outlined (see website section below)
- Peer worker phone support is likely to involve setting up an appointment or appointments. If they wish to, participants can communicate about an issue via text or email rather than a phone call.
- It needs to be made clear that the peer support number is not an emergency number
- People providing and receiving peer support will discuss situations and problems together just as they would in other peer support environment, and together they will look at what actions can be undertaken
- The peer worker will refer back to material covered in relevant sessions when appropriate.
- The peer worker will use the following strategies:
    - **OARS:**
        - **Open Questions**
        - **Affirmations**
        - **Reflections**
        - **Summaries**
    - **Elicit-provide-elicit** when the issue relates to information about HIV or HIV sharing:
        - Information is elicited from the person so the therapist can better understand their attitudes, beliefs, values, and readiness to change. First, the therapist ***elicits*** from the client what they already know (and what they want to know). This helps identify gaps or errors in the client's current knowledge.
        - Then, after asking permission, if the client is interested, the therapist ***provides*** the information that might be helpful to the client, in a neutral manner.

- And finally, after providing the information, the therapist ***elicits*** the client's reaction to the information. Maybe this is something that the client has tried before. Or maybe the client didn't understand what was said, or maybe there is a major barrier to trying the idea you mentioned.

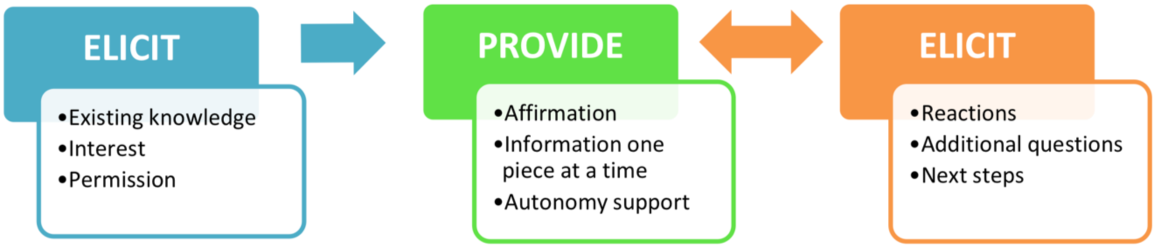

- Record contact with a brief summary.

Possible scenarios that might arise and suggestions of what to do in these situations:

- **The YP has shared, whether planned or unplanned (e.g., the other person guessed or the sharing was accidental)**
  - Ask how it went (what happened, what their thoughts were at the time and after, how they felt at the time and after) (OARS)
  - What they learned
  - What they/the person they shared with would like now, including from you (Elicit)
  - If it did not go well/the recipient had a negative reaction, remind them of what they learned in session 3 – they can check the "after sharing" section in their workbook. E.g. remind them that the reaction says more about them than it does about you, reactions can change over time (and with more education), it doesn't mean future reactions will be negative… (Provide)
  - Ask their response to this (Elicit)

- **Someone has been the recipient of HIV sharing**
  - Ask how it went (what happened, what their thoughts were at the time and after, how they felt at the time and after) (OARS)
  - What they/the person they shared with would like now, including from you (Elicit)
  - Provide information/revert to relevant section of intervention sessions. Consider offering specific advice with permission, e.g., staying calm, providing support, being honest about your feelings (Provide)
  - Ask their response to this (Elicit)

- **The YP is thinking of sharing or wants help with how to share**
    - Explore thoughts and feelings about sharing (OARS) and what they would like, including from you (Elicit)
    - Provide information/revert to relevant section of intervention sessions/revert to proforma (Provide)
    - Identify sharing goal in relation to values and personal sharing rules (session 4)
    - Discuss sharing plan if they are ready to do this (session 4)
    - Ask their response to this (Elicit)

- **The YP wants information about HIV (possibly in relation to sharing their HIV status), e.g., legal information, leaflets, websites.**
    - Ask what information they would like and why (Elicit)
    - Provide relevant information, referring back to session 1 (Provide)
    - Ask their response to this (Elicit)

- **The YP has told someone and that person has told someone else without their permission**
    - Ask what has happened, their thoughts, and how they feel (OARS)
    - What they would like now, including from you (Elicit)
    - Remind them of what they learned in the sessions. For example:
        - Session 3 – tips for how to communicate about HIV sharing may be relevant if the YP wants to communicate their feelings towards that person (Provide)
    - Ask their response to this (Elicit)

- **The YP feels pressured into sharing or pressured into having sex (e.g., their partner wants to have sex and the YP does not want to have sex without sharing; their partner wants to have sex without a condom but the YP does not because of not adhering to their medication)**
    - Ask what has happened, their thoughts, and how they feel (OARS)
    - What they would like now, including from you (Elicit)
    - Remind them of what they learned in the sessions. For example:
        - Session 3 – tips for how to communicate about HIV sharing may be relevant if the YP wants to share their status now or to communicate their feelings about sex (Provide)
    - Ask their response to this (Elicit)

- **The YP is struggling with their mental health**
    - Ask about how they are feeling and thinking, and what they are doing (OARS)
    - Assess risk
    - Identify triggers, when it is less or more of a problem, coping (including available support)
    - If issue is related to HIV sharing, revert to relevant session
    - Ask they would like now, including from you.
    - Contact member of clinical team with participant's permission

- **The young person having a difficult conversation with a family member about wanting to share their diagnosis**
  - Explore their thoughts and feelings about sharing (OARS), their family member's thoughts and feelings about sharing, and what they would like, including from you (Elicit)
  - Offer to talk to family member?
  - Provide information/revert to relevant section of intervention sessions, e.g., coping plan section of session 4 (Provide)
  - Ask their response to this (Elicit)

- **YP hears someone say something stigmatising about HIV, for example a 'joke' or inaccurate information about HIV**
  - Ask what happened, what their thoughts were at the time and after, how they felt at the time and after) (OARS)
  - What they learned
  - What they would like now, including from you (Elicit)
  - Revert to session 3 – they can check the "after sharing" section in their workbook. E.g. remind them that this says more about them than it does about you, and attitudes can change over time (and with more education) (Provide)
  - Ask their response to this (Elicit)
