# Supplementary material for: The HIV Empowering Adults’ Decisions to Share: UK/Uganda (HEADS-UP) Study—A Randomised Feasibility Trial of an HIV Disclosure Intervention for Young Adults with Perinatally Acquired HIV
Source: AIDS Behav. 2024 Mar 15;28(6):1947–64. doi: 10.1007/s10461-024-04294-2 (PMC11161430; doi:10.1007/s10461-024-04294-2)
Supplement: Supplementary file 2 — Supplementary file2 (DOCX 18 KB) [file 10461_2024_4294_MOESM2_ESM.docx]

# Supplemental Material

**Supplemental Table 1. Fixed effects results for aggregated models after multiple imputation on the analysis of Adolescent HIV Disclosure Cognitions and Affect Scale.**

Multiple imputation was conducted using MICE (multiple imputation by chained equations) as implemented in the mice package in R (version 3.15)(1). We imputed individual scale items under fully conditional specification including all items and other predictors (gender, age, country, lifetime HIV disclosure at baseline, condition and timepoint) in the imputation model. The total score of the scale was then computed for each of the imputed datasets and used as dependent variable. We created 100 imputated datasets and used ‘Rubin’s rules’ (2) to pool parameter estimates and compute p-values.

|  | *β* | SE | *t* | *df* | *p* |
| --- | --- | --- | --- | --- | --- |
| (Intercept) | 57.09 | 1.90 | 30.06 | 843.05 | **<0.001** |
| Condition (Intervention) | -3.40 | 2.20 | -1.54 | 2067.94 | 0.12 |
| Country | -2.81 | 1.21 | -2.33 | 1217.10 | 0.02 |
| Gender | 0.25 | 0.88 | 0.28 | 4166.66 | 0.78 |
| LifetimeDisclosure | 3.72 | 0.81 | 4.57 | 11761.11 | **<0.001** |
| Age | 1.72 | 0.96 | 1.78 | 4172.81 | 0.07 |
| Time (follow-up) | 1.10 | 1.94 | 0.57 | 891.34 | 0.57 |
| Time (follow-up) ×  Condition (Intervention) | 4.46 | 2.59 | 1.72 | 1141.08 | 0.09 |

**References**

1. Van Buuren S, Groothuis-Oudshoorn CGM. Mice‘: Multivariate Imputation by Chained Equations in R. Journal of Statistical Software. 2011;45(3):1-67.

2. Barnard J, Rubin BK. Small-Sample Degrees of Freedom with Multiple Imputation. Biometrika. 1999;86(4):948-55.
